# USP36 facilitates esophageal squamous carcinoma progression via stabilizing YAP

**DOI:** 10.1038/s41419-022-05474-5

**Published:** 2022-12-05

**Authors:** Wenhao Zhang, Junwen Luo, Zhaohua Xiao, Yifeng Zang, Xin Li, Yougjia Zhou, Jie Zhou, Zhongxian Tian, Jian Zhu, Xiaogang Zhao

**Affiliations:** 1 Department of Thoracic Surgery, The Second Hospital, Cheeloo College of Medicine, Shandong University, Shandong Province, People’s Republic of China; 2 Department of General Surgery, The Second Hospital, Cheeloo College of Medicine, Shandong University, Shandong Province, People’s Republic of China; 3 Xinxiang Key Laboratory of Tumor Migration and Invasion Precision Medicine, School of Laboratory Medicine, Xinxiang Medical University, Xinxiang, 453003 Henan Province People’s Republic of China; 4 Key Laboratory of Thoracic Cancer in Universities of Shandong, Shandong University, Shandong Province, People’s Republic of China

**Keywords:** Oncogenes, Oesophageal cancer

## Abstract

Esophageal squamous carcinoma (ESCC) is the major subtype of esophageal cancer in China, accounting for 90% of cases. Recent studies revealed that abnormalities in the Hippo/YAP axis are pervasive in ESCC and are recognized as the important driver of ESCC progression. Since the activity of Hippo signaling is controlled by phosphorylation cascade, it is a mystery why the major effector YAP is still over-activated when the cascade is inhibited. Several studies suggested that in addition to phosphorylation, other protein modifications such as ubiquitination also play important roles in manipulating Hippo/YAP signaling activity. Since YAP protein stability is controlled via an appropriate balance between E3 ubiquitin ligases and deubiquitinases, we performed deubiquitinase siRNA screening and identified USP36 as a deubiquitinase significantly related to Hippo/YAP signaling activity and ESCC progression. USP36 expression was elevated in ESCC samples and correlated with poor differentiation. USP36 expression was correlated with YAP protein levels in ESCC samples. Molecular studies demonstrated that USP36 associated with the YAP protein and enhanced YAP protein stability by blocking the K48-linked polyubiquitination of YAP. In conclusion, our study revealed a novel deubiquitinase in regulating Hippo signaling in ESCC, which could be an encouraging drug target for Hippo-driven ESCC.

## Background

Esophageal carcinoma is one of the leading malignancies worldwide [1]. There are more than 4.5 million new cases diagnosed, while half of them are found in China [2]. Esophageal cancer cases in China have distinct pathological characteristics, if compared with western countries. Most esophageal adenocarcinomas in western countries are derived from Barrett’s esophagus, while 90% of esophageal carcinomas are esophageal squamous carcinomas (ESCCs) in China. The incidence of ESCC exhibits high inter-area variations, while most newly diagnosed cases are clustered in northern China regions, such as Shandong and Henan provinces [3]. Several environmental factors, including tobacco and alcohol consumption, have been reported to be associated with the carcinogenesis of esophageal carcinoma, but the detailed mechanism of esophageal cancer progression is still not clear.

Recent genome-wide sequencing data on ESCC samples indicated a link between Hippo signaling and ESCC [4, 5]. It was reported that genomic abnormities, such as mutations and amplifications, in the Hippo pathway were pervasive. Mutations in inhibitory modulators of Hippo signaling, including AJUBA and FATs, was found in ESCC. Although a few studies reported a different requirement of YAP or TAZ in different context, both the gene amplifications of YAP/TAZ were found in ESCC samples [5, 6]. Several molecular biological studies have shown that depletion of YAP and TAZ can inhibit ESSC growth [7]. In view of these conclusions, we propose that targeting Hippo signaling could be an effective strategy for ESCC patients.

Hippo signaling was first found in Drosophila and was further investigated in the context of human organ size control and tissue regeneration [8]. The core components of the Hippo pathway comprise one phosphorylation cascade: the Hippo kinases MST1/2 promote the phosphorylation of LATS1/2 kinases, while phosphorylated LATS1/2 phosphorylates the YAP protein at several serine sites, which later facilitates YAP cytosol retention and proteasome-dependent degradation [9, 10]. If this phosphorylation cascade is not active, unphosphorylated YAP will translocate into the nucleus and combine with several transcription factors, including TEADs, to regulate target gene expression [11]. Activation of the YAP protein has been observed in several human cancers, including ESCC [12–14].

YAP is an important downstream effector of Hippo signaling that functions as a cofactor for various transcription factors, such as TEADs [15]. In addition, YAP protein can also trans-activate other signaling pathways, such as Hedgehog and Wnt signaling [16–18]. Multiple studies revealed that YAP protein expression was elevated in human malignancies, and this up-regulation was correlated with advanced tumor stage and poorer prognosis [19]. Thus, blocking or inhibiting YAP, which subsequently activates the tumor suppressive function of Hippo signaling in human cancer could be a potential therapeutic strategy in human cancers. As YAP is an obvious target assembly of the ubiquitin–proteasome system (UPS) [20], promoting protein degradation could be applied to restore the tumor-inhibitory function of Hippo signaling for ESCC therapeutics. Our previous studies focused on E3 ubiquitin ligases in Hippo signaling in human ESCC [7]. However, the protein stability depends on the balance of E3 ubiquitin ligases and deubiquitinases [21–23], and little is known about deubiquitinases in Hippo signaling and ESCC progression. Our present study revealed that USP36 is an important factor in modulating Hippo signaling activity and ESCC growth. USP36 could interact with the YAP protein and inhibit YAP K48-linked polyubiquitination. Thus, targeting USP36 could be a plausible therapeutic strategy for ESCC patients.

## Materials and methods

### Cell culture

Human ESCC cell lines (KYSE150, EC9706, and ECA109) and HEK293 cells were purchased from Fuheng Biology (Shanghai, China). STR (short tandem repeat) amplification was used for cell line authentication. ECA109, KYSE150, and EC9706 cells were maintained in RPMI-1640 medium (Corning, USA). HEK293 cells were grown in DMEM (Corning, USA). All media contained 1% penicillin/streptomycin (NAM Biotech, China) and 10% fetal bovine serum (FBS; HyClone, USA). All cells were cultured at 37 °C in a 5% CO_2_ humidified atmosphere.

### SiRNA and plasmids

Small interfering RNA (siRNA) was used to knock down specific genes. For the current study, USP36 siRNA or shRNA was synthesized by TSINGKE (China), and the sequences are shown in Supplementary Table 1. RNAiMAX reagent (13778-150, Invitrogen) was used for siRNA transfection. To construct shRNA for USP36 silence, the sequences targeting the back-splicing junction of USP36 were ligated into the Plent-U6-GFP-Puro lentiviral vector. The expression plasmids encoding USP36-Flag and USP36 truncation mutants were formed by PCR and consecutive insertion of the relevant fragment into the pcDNA3.1(+) vector. The Myc-YAP, YAP truncation mutants, HA-UB, HA-K48, HA-K63 and TEAD-reporter plasmids were acquired from our previous study [7]. Plasmids were transfected using Lipofectamine 3000 (L3000-015, Invitrogen).

### Clinical samples

A tissue microarray (TMA) containing 107 ESCC tumorous tissue and adjacent normal tissue samples was generated from the paraffin-embedded tissues of the Second Hospital of Shandong University. All patients underwent esophagectomy with complete resection, and none received any anticancer treatments prior to biopsy collection. The postoperative stage was defined based on the eighth edition of the International Union Against Cancer (UICC) tumor-node-metastasis (TNM) classification criteria. We acquired the ethic permit from Ethics Committee of the Second Hospital of Shandong University.

### RNA isolation and quantitative real-time PCR (qPCR)

Total RNA was extracted with the RNA Easy Fast Tissue/Cell Kit (TIANGEN, China; Cat: #DP451) following the instructions. Reverse transcription was performed using the lnRcute lncRNA First-Strand cDNA Kit (TIANGEN, China, Cat: #KR202). The lnRcute lncRNA qPCR Kit (SYBR Green) (TIANGEN, China, Cat: FP402) was used with SYBR Green to perform quantitative-PCR (QPCR) analysis of mRNA. Then, QPCR was performed using a QuantStudio TM 5 Real-Time PCR System (Thermo Scientific, USA). The 36B4 gene served as the internal control. The 2−∆∆Ct analytic method was employed to calculate the relative expression levels. The primers used in the current study were composed by TSINGKE (China) and are listed in Supplementary Table 2.

### Western blotting

The cells were harvested and lysed with RIPA buffer containing protease and phosphatase inhibitors (Beyotime, China). After quantification, equivalent amounts of protein were separated by 10% SDS–PAGE and transferred to PVDF membranes (Millipore, USA). Then, the membranes were blocked in 5% skim milk for 1 h and incubated with primary antibodies at 4 °C overnight. Afterward, the membranes were washed three times with TBST and incubated with secondary antibodies for 2 h. After being washed three times with TBST, the membranes were probed with an ECL system (Millipore, Cat. No. WBKLS0500), and images were captured using a Tanon 5200 system (Tanon, China). β-Actin served as the internal control. The primary antibodies and secondary antibodies used in the present study are shown in Supplementary Table 3. The full and uncropped western blots were uploaded in Supplementary materials.

### Cell counting Kit-8 (CCK-8) assay

Cell viability was examined with a Cell Counting Kit (CCK-8) assay, after transfection, 3 × 10^3^ cells were plated into 96-well plates. Then, the proliferation level was determined at 0, 24, 48, and 72 h. Ten microliters of CCK-8 kit solution (Dojindo, Japan) with 90 µl medium was placed in each well and then cultured for 2 h. Finally, the optical density (OD) value at 450 nm was determined using a multifunctional enzyme-linked analyzer (BioTek, USA).

### Colony formation assay

For the colony formation assay, after transfection, 1 × 10^3^ cells were plated into 6-well plates and continuously cultured for approximately 2 weeks. After fixation with 4% paraformaldehyde for 15 min and staining with 0.1% crystal violet for 30 min, visible colonies were imaged and counted.

### EdU incorporation assay

For the EdU assay, after transfection, 3 × 10^3^ cells were seeded into 96-well plates and cultured for 24 h. Briefly, the cells were incubated with 100 µl 5-ethynyl-2-deoxyuridine solution (Beyotime, China) for 2 h and then incubated with 4% paraformaldehyde. After washing and permeabilizing the cells with 3% bovine serum albumin (BSA) and 0.3% Triton X-100, 1× Hoechst 33342 reaction solution was used to stain nuclei. Finally, images were captured using a fluorescence microscope. The percentage of EdU-positive cells was calculated as follows: (EdU-stained cells/Hoechst-stained cells) ×100%. The experiment was performed in triplicate.

### Wound healing assay

For the wound healing assay, After transfection, 5 × 10^4^ cells suspended in 110 µL medium were added to each well containing a culture insert (Ibidi, Germany) and incubated overnight. When the cells reached 95–100% confluence, a scratch was made across the insert to create a wound. After being washed with 1×PBS twice, the cells were cultured with serum-free medium. Images were captured using an inverted microscope (LEICA, Germany) at 100× magnification every 6 h or 12 h. The rate of wound healing was analyzed with ImageJ software. The experiment was performed in triplicate.

### Transwell assay

Uncoated transwell chambers (8 µm pore size; Corning, USA) were used for the migration assay, whereas 50 µl Matrigel (1:10; BD Biosciences, USA) was used to precoat the upper surface of the transwell chambers in the invasion assay. First, the upper chamber was seeded with 5 × 10^4^ cells resuspended in 200 µl serum-free medium, and the lower chamber was seeded with 600 µl medium supplemented with 10% FBS. After incubation for 48 h, the cells that penetrated the lower surface of the membranes were incubated with 4% paraformaldehyde and dyed with hematoxylin. Finally, cells from three randomly selected fields were counted. The experiment was performed in triplicate.

### 3D culture assay

According to the protocol, 2× medium and 1% agar were mixed at a ratio of 1:1 (with 10% FBS and 1% penicillin/streptomycin), and then 2 ml medium was added to each well of a 6-well plate as the basal medium. The plate was incubated for 4 h at room temperature to set the gel. The upper cell culture medium contained 2 × medium and 0.6% agar mixed at a ratio of 1:1 (with 1% penicillin/streptomycin and 10% FBS). Cells were suspended in the upper medium and added to 6-well tissue culture plates with basal medium. After the top layer solidified at room temperature, the plate was cultured in a CO_2_ incubator at 37 °C. After culturing for 14 days, spheres with diameters of >40 µm were counted.

### Dual-luciferase reporter assay

ECA109, KYSE150, or EC9706 cells were seeded in 24-well plates. TEAD luciferase reporter plasmid along with Renilla expression plasmid and the indicated plasmid were cotransfected into ESCC cells. After transfection for 24 h, luciferase activity was determined by a dual luciferase assay kit (Promega, USA) according to the manufacturer’s protocol. The experiments were executed in triplicate.

### Apoptosis assay

Cells were transfected with siRNA or plasmid. At 24 h or 48 h after transfection, the cells were marked with propidium iodide (PI) and annexin V. Fluorescence intensity was determined using a CytoFLEX flow cytometer. The experiments were executed in triplicate.

### Immunohistochemistry (IHC)

Tissues were incubated with 4% paraformaldehyde, embedded in paraffin, and then cut into slices using a Semi Automated Paraffin Microtome S700 (RWD, USA). IHC staining was performed with an IHC kit (Zsbio, Beijing, China) according to the manufacturer’s protocol. We used primary antibodies against YAP and USP36. After incubations with the primary and secondary antibodies, the immunocomplex was revealed with DAB, and the nuclei were counterstained with hematoxylin. We then used a NanoZoomer Digital Pathology scanner (NanoZoomer S60, HAMAMATSU, Japan) to obtain pictures. The result was evaluated by the mean of Integrated optical density (IOD) via ImageJ Software. The primary antibodies used in the IHC analysis are also shown in Supplementary Table 3.

### Immunofluorescence assay

Cells on the coverslips were incubated with 4% paraformaldehyde for 10 min, permeabilized with 0.3% Triton X-100 for 10 min, and blocked with 5% BSA in PBS for 1 h. After that, the cells were incubated with primary antibodies against USP36 and YAP at 4 °C overnight. After washing with PBS, the cells were then incubated with a fluorescence-conjugated secondary antibody (Supplementary Table 3) and counterstained with DAPI (Beyotime). Images were captured after staining with anti-fade DAPI solution using a confocal laser-scanning microscope (LSM 800, Zeiss, Germany). Images were subsequently processed and assembled using ImageJ.

### Protein stability assay

Approximately 10^5^ ECA109 cells were seeded into 12-well plates and transfected with USP36 siRNA or siControl. After 48 h, the cells were processed with 100 μM cycloheximide (CHX) (C7698, Sigma) for the indicated times. Samples were used for western blotting to evaluate YAP、TAZ degradation.

### Coimmunoprecipitation (Co-IP) assay

Cells were transfected with the indicated plasmids or subjected to the indicated treatment for 48 h and were then collected and lysed in 1 ml of NP-40 buffer (Beyotime, China) with complete protease inhibitor cocktail (MCE, HY-K0010) on ice for 30 min. The cell lysate was centrifugation at 12,000 rpm for 15 min at 4 °C. Fifty microlitres of lysate supernatant were taken as input, and the rest was incubated overnight with Protein A/G Magnetic Beads (MCE, HY-K0202) and the anti-YAP (Proteintech, 13584-1-AP), anti-USP36 (Proteintech, 4783-1-AP), anti-Flag-tag (Cell Signaling Technology, 14793S) or anti-Myc-tag (Cell Signaling Technology, 2278S) antibody at 4 °C on a rotating device. The corresponding IgG (Beyotime) was used as the negative control. Next, the magnetic beads were washed 4 times for 5 min each with cold NP-40 buffer and boiled for 10 min in 30 µl of 2 × loading buffer. After centrifugation, the supernatant was collected and subjected to western blot analysis.

### Polyubiquitination detection assay

To directly detect YAP polyubiquitination in cell extracts, HEK293 cells were transfected with the Ub, K48 Ubi, and K63 Ubi plasmids together with the Flag-USP36 plasmid and Myc-YAP or vector. After 48 h, the cells were processed with 20 μM MG132 (MCE, HY-13259) for 6 h. Total protein was then collected, and lysates were precleared with IgG and 30 μl of protein A + G Agarose (Beyotime, P2055) for 2 h. The supernatant was harvested and immunoprecipitated with an anti-Myc antibody. Western blotting with an anti-HA antibody was performed to detect YAP polyubiquitination.

### In vivo tumorigenesis and metastasis assay

For the in vivo tumorigenesis experiment, BALB/c nude mice (Vital River, Beijing, China) (4 weeks, female) were injected with approximately 4 × 10^6^ cells. Tumor formation in nude mice was supervised over 30 days. The tumor volume was computed by the formula: tumor volume = 0.5 × length × width^2^. For in vivo metastasis assays, BALB/c nude mice (4 weeks, female) were injected with approximately 2 × 10^6^ cells intravenously through the tail vein. The mice were killed 8 weeks after injection. Tumor nodules on the lung surface were identified and counted. The lungs were embedded in paraffin. Subsequently, the tissue was stained with H&E to observe the structure.

### RNA sequencing and data analysis

Three biological replicates were prepared for the RNA-seq library and RNA-Seq analysis. Total RNA was extracted with TRNzol Universal Reagent (TIANGEN, DP424), RNA integrity was assessed using the RNA Nano 6000 Assay Kit of the Bioanalyzer 2100 system (Agilent Technologies, CA, USA). For each repeat, equal amounts of RNA were pooled for RNA-Seq library construction. Construction of the RNA-seq library and RNA-seq was performed by Novogene Company (Beijing, China). Differentially expressed genes (DEGs) were analyzed using DESeq2 packages in R language. Genes with log2(fold change) > 1 and FDR < 0.05 were regarded as notable differentially expressed genes. Kyoto Encyclopedia of Genes and Genomes (KEGG) [24] pathway enrichment analysis was executed using the cluster Profiler package in R language. The RNA sequencing data were deposited into the Gene Expression Omnibus (GEO) [25] database (accession number: GSE206845). Gene set enrichment analysis (GSEA) [26, 27] was executed using GSEA software (http://www.broadinstitute.org/gsea).

### TCGA data download

The expression data of ESCC patients were downloaded from TCGA data portal. To analyze the expression of USP36 and Hippo signaling target genes, the expression data of ESCC patients were downloaded from the GEO database. A volcano plot was generated using the OmicStudio tool. The heatmap plot was generated using the Morpheus tool at https://software.broadinstitute.org/morpheus/.

### Statistical analysis

Statistical analyses were conducted with GraphPad Prism 8.0.1 (GraphPad, USA). Differences between groups were analyzed by Student’s t test for comparisons between two groups or one-way analysis of variance (ANOVA) for multiple comparisons. The χ^2^ test was applied for categorical variables. The correlation of measurements was determined using Pearson’s correlation analysis. Measurement data are presented as the mean ± standard deviation (SD), and *P* < 0.05 was considered to indicate a significant difference.

## Results

### USP36 expression is elevated in human esophageal carcinoma and positively correlated with Hippo/YAP signaling activity according to RNA sequencing analysis

**Fig. 1:**
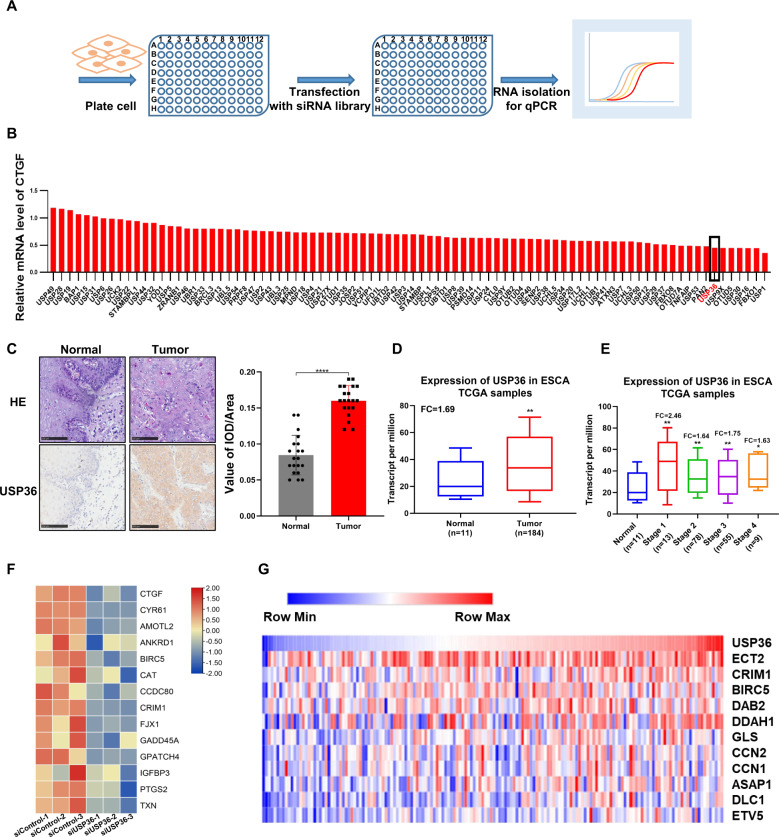
The possible regulation between USP36 and Hippo pathway in ESCC. **A** Diagram of the screening procedure used to identify deubiquitinases that modulate Hippo signaling in HEK293 cells. **B** The relative expression level of CTGF(NM_001901.4) in HEK293 cells transfected with deubiquitinases in the screening library. The RT-qPCR results were normalized to 1. USP36 is marked in red. **C** USP36 expression was markedly increased in 20 ESCC tissues compared with adjacent nontumor tissues as analyzed by IHC. Scale bar, 100 µm. Integrated optical density (IOD) values for IHC staining between normal and tumor tissues are presented in the histogram. USP36 antibody was verified for IHC by immunofluorescence in Supplementary Fig. 1A. **D** The expression level of USP36 in ESCC tumor samples versus normal samples in TCGA was shown in the histogram. **E** Expression of USP36 in ESCC between normal tissues and tumors tissues of different stages is presented in the histogram. **F** The heatmap showed differentially expressed Hippo pathway-related genes whose expression was seriously decreased by siUSP36 in ECA109 cells. **G** Heatmap for USP36 and Hippo signaling pathway target gene expression in TCGA ESCC datasets (in the diagram, red denotes high gene expression levels, blue denotes low gene expression levels). The quantification of the positive correlation between USP36 and the YAP and TAZ target genes is shown in Supplementary Fig. 1D. ****P < 0.0001.

We first performed deubiquitinase siRNA screening to identify novel deubiquitinases for the Hippo pathway. HEK293 cells were used as the model, since they were widely used in Hippo studies and are easily transfected. In addition, CTGF (NM_001901.4) expression was measured as a readout to demonstrate Hippo signaling activity (Fig. 1A). The data identified several reported deubiquitinases, including UBTD1 [28], in our screening. Interestingly, we also uncovered several novel hits, such as USP36 (Fig. 1B). This might indicate important roles of USP36 in Hippo signaling. We further analyzed the protein level of USP36 in human ESCC specimens. The immunohistochemistry data demonstrated that USP36 was increased in 20 ESCC compared with normal esophageal tissues (Fig. 1C). Western blot showed the total amount of USP36 protein was increased in tumor vs matched normal tissues of 8 ESCC patients (Fig. S1C). We further explored the USP36 mRNA level in esophageal cancer patients. The public available TCGA data implicated that USP36 expression was conspicuously upregulated in esophageal cancer samples compared with normal esophageal tissues (Fig. 1D), while USP36 expression was increased in esophageal cancer samples of all stages (Fig. 1E). We further silenced USP36 in ECA109 cancer cells for RNA sequencing analysis. The volcano plot and KEGG pathway analysis showed that USP36 depletion could affect several oncogenic pathways, such as the TNF pathway, MAPK pathway and NFKB signaling, in addition to the Hippo pathway (Fig. S1D–F). The heat map of gene expression after USP36 depletion showed that a group of YAP target genes were inhibited by siUSP36 in ECA109 cells (Figs. 1F, S1B). Finally, by analyzing the expression of USP36 and YAP target genes from the TCGA database, we observed that USP36 was positively correlated with a cluster of YAP target genes in esophageal cancer (Figs. 1G, S1G).

### USP36 is required for the activity of HIPPO/YAP axis in human ESCC

**Fig. 2:**
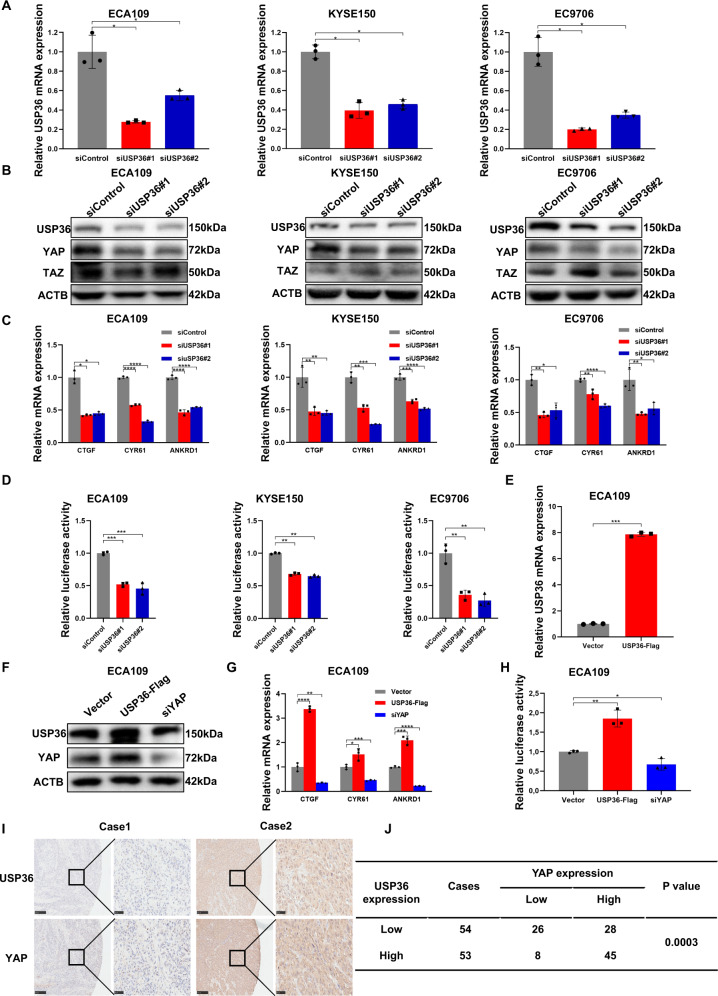
USP36 positively regulates the Hippo pathway in ESCC cell lines. **A** qPCR analysis of USP36 mRNA levels to validate knockdown efficiency in ECA109, KYSE150, and EC9706 cells. **B** Western blotting results showed that USP36 depletion decreased YAP protein levels in ECA109, KYSE150, and EC9706 cells transfected with two independent siRNA targeting USP36 for 48 h, respectively, however, did not affect TAZ protein levels. **C** qPCR results showed that USP36 depletion decreased Hippo target gene expression in ECA109, KYSE150, and EC9706 cells transfected with two independent siRNA targeting USP36 for 48 h, respectively. **D** USP36 depletion inhibited TEAD response element activity in ECA109, KYSE150, and EC9706 cells. Cell lines were transfected with siControl or siUSP36. After 24 h, the cells were transfected with TEAD luciferase reporter plasmids. After another 24 h, the cells were harvested for luciferase activity analysis. **E** qPCR analysis of USP36 mRNA levels to validate overexpression efficiency in ECA109 cells stable expressing USP36-flag or lentiviral vector. **F** Western blotting results showed that USP36 overexpression increased YAP protein levels in ECA109 cells stable expressing USP36-flag or lentiviral vector and siYAP decreased YAP protein levels. **G** qPCR results showed that USP36 overexpression increased Hippo target gene expression in ECA109 cells stable expressing USP36-flag or lentiviral vector, which was decreased by siYAP. **H** USP36 overexpression increased TEAD response element activity in ECA109 cells stable expressing USP36-flag or lentiviral vector, which was decreased by siYAP. **I** High USP36 expression was highly correlated with increased YAP levels in 107 human ESCC samples analyzed by IHC staining. Scale bar, 250 µm (left), 50 µm (right). **J** Statistical analysis of the correlation of USP36 with YAP expression in 107 human ESCC tumor samples. *P < 0.05, **P < 0.01, ***P < 0.001,****P < 0.0001.

We further utilized the ECA109, KYSE150, and EC9706 cell lines to explore the effect of USP36 on Hippo signaling. The qPCR assay showed that USP36 could be effectively silenced via two separate siRNAs (Fig. 2A). The immuno-blotting data indicated that USP36 depletion reduced YAP protein levels in ECA109, KYSE150, and EC9706 cells, but did not affect TAZ protein levels (Fig. 2B). Moreover, some Hippo pathway components (LATS1, LATS2, MST1/2) and p-YAP (ser127) were not affected (Fig. S2A, B). We further analyzed Hippo target gene expression after USP36 depletion; this alteration inhibited the expression of Hippo target genes, including CTGF (NM_001901.4) and CYR61, in ECA109, KYSE150, and EC9706 cells (Fig. 2C). The luciferase reporter assay demonstrated that USP36 inhibition suppressed TEAD response element activity in ECA109, KYSE150 and EC9706 cells (Fig. 2D). We further overexpressed USP36 in ECA109 cells via a lenti-virus system. We first confirmed that USP36 was overexpressed with high efficiency (Fig. 2E). The western blotting data showed that USP36 overexpression enhanced YAP protein levels in ECA109 cells (Fig. 2F). The qPCR data showed that USP36 overexpression enhanced the expression of Hippo target genes, including CTGF (NM_001901.4) and CYR61 (Fig. 2G). The luciferase reporter assay showed that USP36 overexpression enhanced TEAD response element activity in ECA109 cells (Fig. 2H). We subsequently evaluated the expression of USP36 in ESCC samples. According to immunohistochemistry analysis of 107 samples, high USP36 protein expression was correlated with poor cancer differentiation (*P* = 0.007, Table 1). We also observed that the expression of USP36 was positively correlated with that of the YAP protein (*P* < 0.001; Fig. 2I, J).

**Table 1:** Clinicopathological correlation of USP36 expression in ESCC.

Clinicopathologic characteristics	Cases (*n* = 107)	USP36 expression		
		Low (*n* = 54)	High (*n* = 53)	*P*-value
Gender				
Male	87	43	44	0.8049
Female	20	11	9	
Age (years)				
<60	35	18	17	>0.9999
≥60	72	36	36	
Differentiation				
Low	27	20	7	0.007**
Mid and high	80	34	46	
Clinical stage				
I+II	42	23	19	0.554
III+IV	65	31	34	
T stage				
T1 + T2	23	12	11	>0.9999
T3 + T4	84	42	42	
N stage				
No	47	24	23	>0.9999
Yes	60	30	30	

*P < 0.05, **P < 0.01, ***P < 0.001.

The correlation analysis between USP36 and the clincial pathological characteristics in 107 ESCC samples.

Statistical significance (*P* < 0.05) is shown in bold.

### USP36 is required for ESCC cell progression

**Fig. 3:**
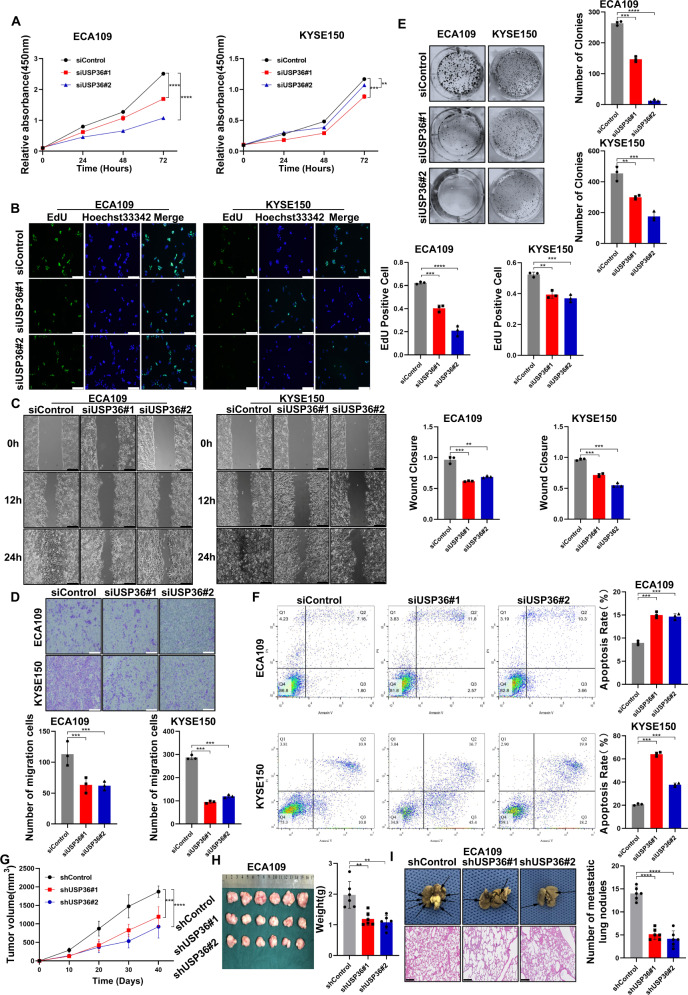
USP36 depletion inhibited ESCC cell progression via Hippo/YAP signaling. **A** Cell viability was determined by CCK8 assay in ESCC cell lines transfected with siControl or two independent USP36 siRNA. CCK-8 assay showed that USP36 depletion inhibited the proliferation of ECA109 and KYSE150 cells. **B** EdU assay to show the cell proliferation of ESCC cell lines transfected with siControl or two independent USP36 siRNA. ECA109 and KYSE150 cells were labeled with EdU. Right panel showed the quantification of EdU results. Green showed EdU-positive cells; blue showed cell nuclei; scale bar, 250 µm. **C** Wound healing assay of ESCC cell lines migration capability following transfected with siControl or two independent USP36 siRNA. The migration assay showed that depletion of USP36 suppressed the migration of ECA109 and KYSE150 cells. Scale bar, 250 µm. Right panel showed quantification of wound healing results. **D** Transwell assay of ECA109 and KYSE150 cells transfected with siControl or two independent USP36 siRNA, respectively. Depletion of endogenous USP36 markedly decreased cell migration. Right panel showed the quantification of transwell assay results. Scale bar, 250 µm. **E** Colony formation of ECA109 and KYSE150 cells transfected with siControl or two independent USP36 siRNA, respectively, showed that USP36 depletion inhibited proliferation. Right panel showed quantification of colony formation assay results. Scale bar, 250 µm. **F** Representative plots (left panel) of apoptosis ECA109 and KYSE150 cells transfected with siControl or two independent USP36 siRNA, respectively. USP36 depletion promoted the apoptosis of ECA109 and KYSE150 cells. Quantitative summary (right panel) of apoptosis analysis of FACS. **G** Quantitative tumor volumes from NSG mice injected with control or USP36-depleted ECA109 cells were measured at indicated time points. Representative image is in Fig. S2J. **H** Representative image of tumor derived from NSG mice injected with control or USP36-depleted ECA109 cells was followed as indicated. Right panel showed quantitative tumor weight of tumors. **I** Representative image of in vivo lung metastasis of indicated ECA109 cells were indicated. The black arrow denoted a pulmonary metastatic nodule. Right panel showed quantitative analysis of tumor metastasis. Scale bar, 250 µm. **P < 0.01, ***P < 0.001, ****P < 0.0001.

We further examined the function of USP36 in ESCC phenotypes. We firstly checked the effect of USP36 in cell proliferation via CCK-8 assay and EdU assay. The CCK-8 is a nonradioactive reagent, which is sensitive to colorimetric measurement for viable cell numbers, while EdU is a thymidine analog, which be incorporated into the DNA of the proliferation cells. The CCK-8 assay showed that USP36 depletion suppressed cancer cell proliferation in ECA109, KYSE150, and EC9706 cells (Figs. 3A, S2C). This conclusion was strengthened by the EdU incorporation assay, which showed that USP36 silencing noticeably decreased the number of EdU-positive ECA109, KYSE150, and EC9706 cells (Figs. 3B, S2D). In the wound-healing assay, we found that USP36 depletion suppressed wound closure speed in ECA109, KYSE150, and EC9706 cells (Figs. 3C, S2F). The Trans-well assay showed that USP36 knockdown inhibited cell migration capacity in ECA109, KYSE150, and EC9706 cells (Figs. 3D, S2E), while the colony formation assay demonstrated that USP36 silencing could significantly reduce the number of colonies of ECA109, KYSE150 and EC9706 cells (Figs. 3E, S2G). We further explored the effect of USP36 on cell death. Propidium iodide (PI) staining coupled with Annexin V staining indicated that USP36 depletion facilitated cancer cell apoptosis in ECA109, KYSE150 and EC9706 cells (Figs. 3F, S2H). This conclusion was strengthened by the western blot of caspase3 (cleaved), which showed that USP36 silencing noticeably increased caspase3 (cleaved) protein levels (Fig. S2I). The 3D culture assay indicated that USP36 was required for sphere formation in ECA109, KYSE150, and EC9706 cells (Fig. S2J). We further evaluated the effect of USP36 in vivo. The xenograft model demonstrated that USP36 depletion significantly reduced ESCC tumor growth in vivo (Figs. 3G, H, S2K). In the in vivo metastasis assay, USP36 depletion significantly reduced lung metastasis in ECA109 cells (Fig. 3I). All these data showed that USP36 is essential for ESCC cell survival and progression.

### USP36 overexpression enhances ESCC cell progression

**Fig. 4:**
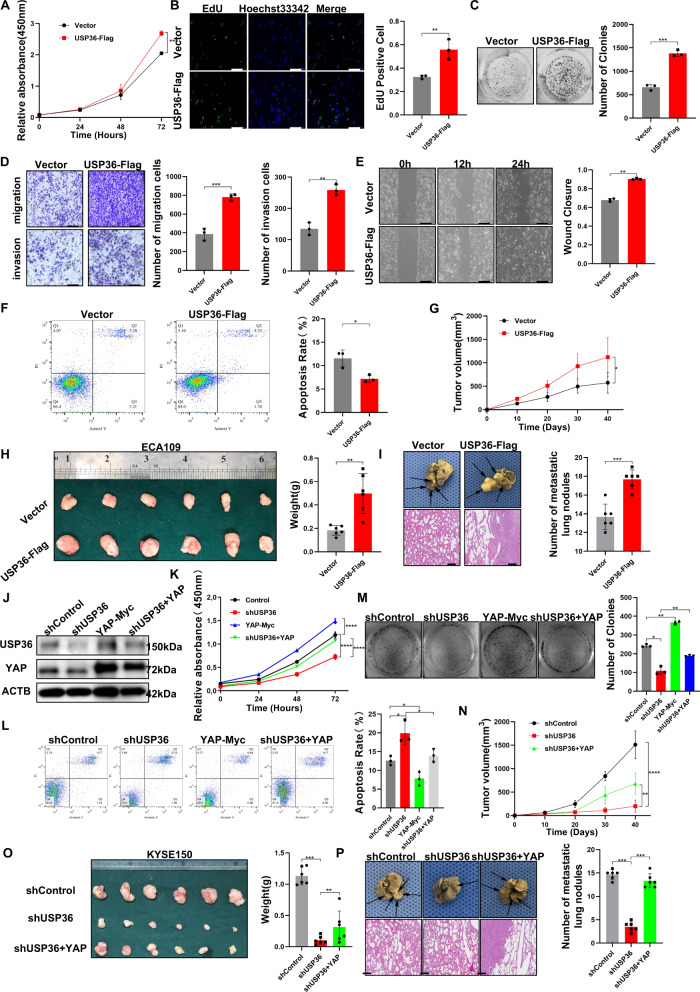
USP36 depletion suppressed ESCC progression through Hippo/YAP signaling, and this effect was attenuated by YAP overexpression. **A**–**I** ECA109 cells were transduced with lentiviruses expressing vector or USP36-Flag. **A** CCK-8 assay showed that overexpression of USP36 increased the proliferation ability of ECA109 cells. **B** EdU assay to showed the cell proliferation of ECA109 cells. ECA109 cells were labeled with EdU. Right panel showed quantification of EdU results. Green showed EdU-positive cells; blue showed cell nuclei; scale bar, 250 µm. **C** Colony formation showed that overexpression of USP36 activated proliferation. Right panel showed the quantification of colony formation assay results. **D** Transwell assays showed that overexpression of USP36 increased cell migration and invasion in ECA109 cells. Right panel showed the quantification of transwell assay results. Scale bar, 250 µm. **E** Overexpression of USP36 markedly increased cell migration, as shown by a wound healing assay. Right panel showed quantification of wound healing results. Scale bar, 250 µm. **F** Overexpression of USP36 inhibited apoptosis in ECA109 cells. Quantitative summary (right panel) of apoptosis analysis of FACS. **G**, **H** The overexpression of USP36 increased ESCC tumor growth in vivo. Tumor growth and tumor weight differed significantly in mice. Representative image was in Fig. S3C. **I** Overexpression of USP36 increased the metastatic ability of ECA109 cells in vivo. The black arrow denoted the pulmonary metastatic nodule. Right panel showed a quantitative analysis of tumor metastasis. Scale bar, 250 µm. **J**–**P** KYSE150 cells were transduced with lentiviruses expressing control or shRNA targeting USP36, following with YAP overexpression. **J** KYSE150 cells were subjected to immunoblotting as indicated. USP36 depletion decreased the YAP protein level, and this effect was attenuated by YAP overexpression. **K** Cell growth of KYSE150 cells were measured by CCK-8 assay showed that USP36 depletion inhibited the proliferative ability, which could be rescued by YAP overexpression. **L** Apoptosis of indicated KYSE150 cells were measured by FACs. USP36 depletion promoted apoptosis in KYSE150 cells, and this effect could be attenuated by YAP overexpression. Quantitative summary (right panel) of apoptosis analysis of FACS. **M** Colony formation assay showed that USP36 depletion inhibited proliferation, which could be rescued by YAP overexpression in KYSE150 cells. Right panel showed quantification of colony formation assay results. **N**, **O** YAP overexpression rescued the growth of xenograft tumors derived from cells with USP36 depletion in vivo. Representative image (**O**) and quantitative tumor weight (Right panel) were shown. **P** Overexpression of YAP suppressed the metastatic ability of cells with USP36 depletion in vivo. The black arrow denoted the pulmonary metastatic nodule. Right panel showed quantitative analysis of tumor metastasis. Scale bar, 250 µm. *P < 0.05, **P < 0.01, ***P < 0.001, ****P < 0.0001.

We further explored the effects of USP36 overexpression in ESCC cells. The CCK-8 assay showed that USP36 overexpression facilitated cell proliferation in ECA109 cells (Fig. 4A), which was also observed in the EdU incorporation assay (Fig. 4B). The colony formation assay showed that USP36 overexpression noticeably increased the number of ECA109 cell colonies (Fig. 4C). The transwell assay demonstrated that USP36 overexpression could further promote the migration and invasion of ECA109 cells (Fig. 4D). In the wound healing assay, we found that USP36 overexpression increased the wound closure rate of ECA109 cells (Fig. 4E). The apoptosis assay demonstrated that USP36 overexpression could prevent cell death in ECA109 cells (Fig. 4F), which was also observed in the western blot, caspase3(cleaved) protein levels was noticeably decreased by USP36 silencing (Fig. S3A). The 3D culture assay indicated that USP36 overexpression could increase the sphere formation capacity of ECA109 cells (Fig. S3B). We subsequently investigated the effect of USP36 in vivo. The xenograft model showed that USP36 overexpression significantly promoted ESCC tumor growth in vivo (Figs. 4G, H, S3C). In the in vivo metastasis assay, USP36 overexpression significantly enhanced the lung metastasis of ECA109 cells (Fig. 4I).

### USP36 facilitates ESCC progression via the Hippo/YAP axis

Since we proved that USP36 is required for ESCC progression and Hippo/YAP activation, we carried out further rescue experiments to test whether USP36 modulates cancer progression through YAP. We depleted USP36 in KYSE150 cells and then overexpressed YAP. The western blot assay showed that the reduction in YAP protein level induced by USP36 depletion could be reversed by YAP overexpression (Fig. 4J). The qPCR data showed that YAP overexpression could rescue Hippo target gene expression, which was reduced by USP36 silencing (Fig. S3D–F). In addition, the luciferase assay indicated that USP36 depletion could inhibit YAP-TEAD luciferase activity, which could be partially rescued by YAP overexpression (Fig. S3G). The CCK-8 assay indicated that USP36 depletion suppressed ESCC growth, while YAP overexpression partially attenuated this growth inhibition effect (Fig. 4K). The EdU staining assay indicated that YAP overexpression could at least partially reverse the decrease in the number of EdU-positive cells induced by USP36 knockdown (Fig. S3H). PI/Annexin V double staining coupled with FACS analysis showed that USP36 depletion enhanced cell apoptosis, which could be partially reversed by further YAP overexpression in KYSE150 cells (Fig. 4L), this conclusion was observed by the western blot of caspase-3(cleaved), which showed that YAP overexpression noticeably decreased caspase-3(cleaved) protein levels (Fig. S3J). In the colony formation assay, the reduced clone numbers caused by USP36 depletion could be partially rescued by YAP overexpression (Fig. 4M). The transwell assay showed that the impaired migration and invasion capacity induced by USP36 depletion could be partially recovered by YAP overexpression in KYSE150 cells (Fig. S3I). The 3D culture assay indicated that USP36 depletion could inhibit sphere formation capacity in KYSE150 cells, which could be partially rescued by YAP overexpression (Fig. S3K). In the wound healing assay, YAP overexpression also restored the cell migration rate, which was reduced by USP36 depletion in KYSE150 cells (Fig. S3L). In the xenograft mouse model, USP36 depletion inhibited tumor growth, and this effect was reversed by YAP overexpression in KYSE150 cells (Figs. 4N, O, S3M). In the in vivo metastasis assay, USP36 depletion significantly reduced lung metastasis, which was partially attenuated by YAP overexpression in KYSE150 cells (Fig. 4P). All these data showed that USP36 promotes ESCC progression via YAP.

### USP36 stabilizes the YAP protein by inhibiting the K48-linked polyubiquitination of YAP

**Fig. 5:**
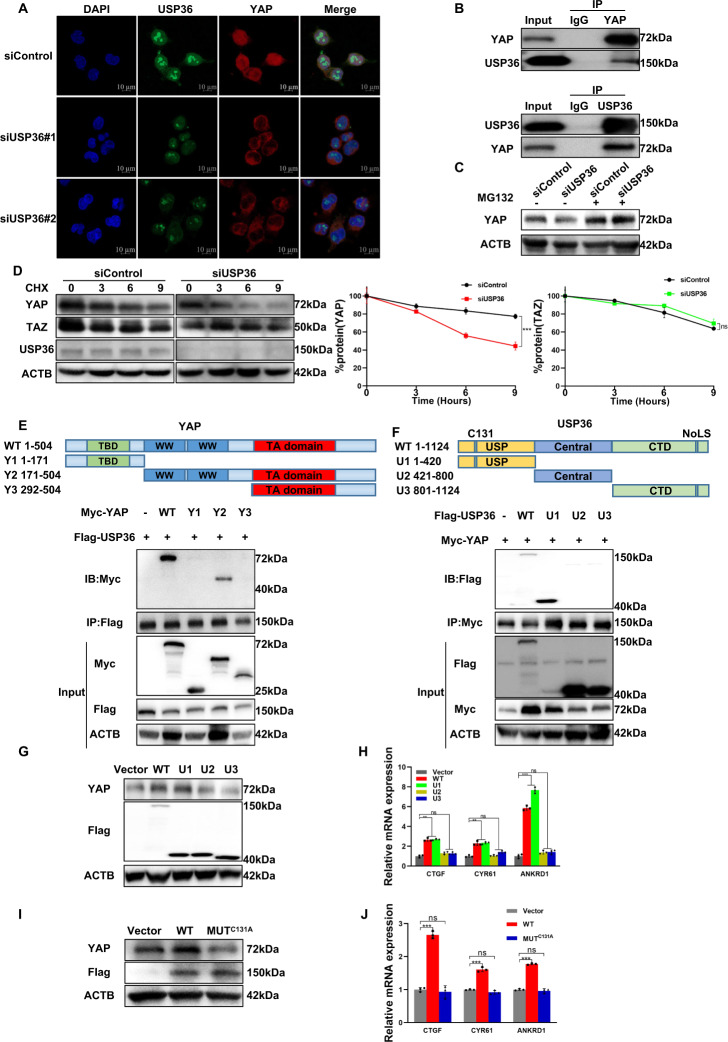
USP36 expression is positively correlated with YAP expression and interacts with YAP in ESCC cells. **A** Immunostaining demonstrated the colocalization of USP36 (green) and YAP (red) in ECA109 cells. Blue denoted cell nuclei. Scale bar, 10 µm. **B** Representative immunoblots to show the interaction between USP36 and YAP as assessed by immunoprecipitation (IP) for USP36 or YAP, compared to isotype control (IgG). **C** Control or USP36-slienced ECA109 cells were treated with MG132 (10 μM) for 24 h. YAP and USP36 protein were measured by immunoblotting. Depletion of USP36 decreased YAP protein levels, and this effect could be attenuated by MG132. **D** USP36 depletion decreased the YAP protein half-life in ECA109 cells. The cells were treated with 100 µmol/L CHX for the indicated time before being collected for western blot assays. Quantitative analysis of halftime of YAP and TAZ protein in (**D**). **E** Representative immunoblots to show the interaction between USP36 and WT or truncated YAP as indicated assessed by immunoprecipitation (IP) with USP36 (Flag). YAP binded to USP36 at its WW domain. (Top panel) Schematic diagram of vectors expressing Myc-tagged wild-type or serial deletion mutants of YAP. (Bottom panel) The WW domain of YAP interacted with USP36. **F** Representative immunoblots to show the interaction between YAP and WT or truncated USP36 as indicated assessed by immunoprecipitation (IP) with YAP (anti-Myc). USP36 binded to YAP at its UPS domain. (Top panel) Schematic diagram of vectors expressing Flag-tagged wild-type or serial deletion mutants of USP. (Bottom panel) The USP domain of USP36 interacted with YAP. **G**, **H** ECA109 cells were transfected with vector, WT, or truncated USP36 and then subjected to immunoblotting to examine YAP protein level or qPCR to examine YAP target gene expression. The USP domain was essential for USP36 to maintain YAP protein levels and YAP target gene expression. **I**, **J** ECA109 cells were transfected with vector, WT, or USP36 mutant(C131A) and then subjected to immunoblotting to examine YAP protein level or qPCR to examine YAP target gene expression. The C131A mutation of USP36 impaired its ubiquitination activity and reduced the ability of USP36 to maintain YAP protein levels and YAP target gene expression in ECA109 cells. **P < 0.01, ***P < 0.001.

Since USP36 modulates the Hippo/YAP axis and ESCC progression, we further investigated the potential mechanism. Immunostaining indicated that both the YAP protein and USP36 protein were enriched in the nucleus, while USP36 depletion significantly reduced YAP staining signals in the nucleus (Fig. 5A), this conclusion was observed by the western blot of the distribution of YAP in nucleo-cytoplasmic extracts. (Fig. S3N). The endogenous immunoprecipitation assay indicated that USP36 could combine with YAP in ESCC cells (Fig. 5B). Since YAP degradation relies on the effect of the proteasome, we utilized the proteasome inhibitor MG132. USP36 depletion decreased YAP protein levels, while MG132 treatment diminished this decrease in YAP protein levels (Fig. 5C). The protein half-life assay demonstrated that USP36 reduction impaired YAP protein stability in ESCC cells, which wasn’t observed in TAZ protein (Fig. 5D). We further investigated the interaction domains between USP36 and YAP. The YAP protein is composed of the TEAD binding (TB) domain, WW domain and transactivation (TA) domain, while the USP36 protein is composed of the USP (deubiquitinase) domain, central domain and C-terminal domain (CTD) (Fig. 5E, F). We made deletion constructs targeting these domains for interaction analysis, which revealed that the WW domain was necessary for the binding of YAP to USP36, while the USP domain was required for the association of USP36 with YAP (Fig. 5E, F). We further overexpressed the deletion domains of USP36 to determine their impact on the Hippo/YAP axis. The western blotting data showed that full-length USP36 and the USP domain could stabilize the YAP protein (Fig. 5G). The qPCR data showed that the USP domain was necessary and sufficient to increase endogenous Hippo target gene expression (Fig. 5H). Since previous studies indicated that cysteine 131 is critical for USP36 to exert its deubiquitinase function [29], we made a mutation variant and transfected it into HEK293 cells. The western blotting data indicated that the C131 site of USP36 was required for its regulation of YAP protein expression (Fig. 5I). The qPCR data indicated that the C131A mutant form abolished the effect of USP36 on Hippo target gene expression (Fig. 5J).

**Fig. 6:**
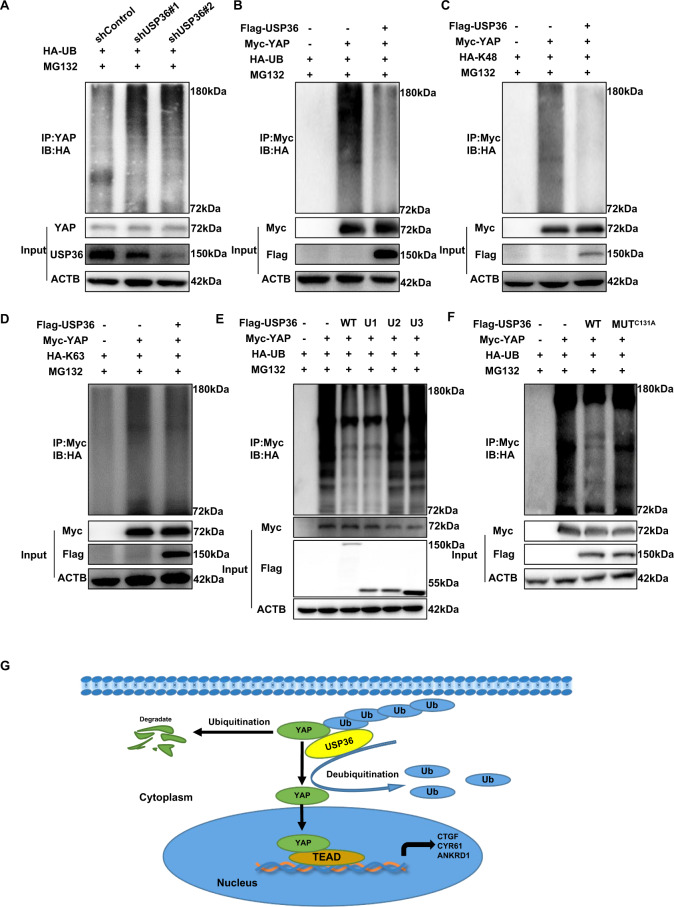
USP36 modulates YAP stability in a ubiquitination-dependent manner. **A** The ubiquitination assay was performed with shUSP36 in ECA109 cells. After 48 h, the cells were treated with MG132 (20 µM) for 6 h. The ubiquitination assay demonstrated that depletion of USP36 promoted endogenous YAP total poly-ubiquitination in ECA109 cells. **B**–**D** HEK293 cells were co-transfected with YAP and USP36 constructs as indicated. After 48 h, the cells were treated with MG132 (20 µM) for 6 h. Global ubiquitination (**B**) K48- and K63-linked ubiquitination (**C**, **D**) of YAP were measured by ubiquitination assay. The ubiquitination assay demonstrated that USP36 inhibited YAP total poly-ubiquitination and K48-linked ubiquitination in HEK293 cells, while had little effect on YAP K63-linked ubiquitination. **E** Global ubiquitination of YAP in HEK293 cells transfected with WT or USP36 mutants was measured by ubiquitination assay. After 48 h, the cells were treated with MG132 (20 µM) for 6 h. The USP domain was essential for USP36 to regulate ubiquitination of YAP. **F** Global ubiquitination of YAP in HEK293 cells transfected with WT or USP36 mutant (C131A) was measured by ubiquitination assay. After 48 h, the cells were treated with MG132 (20 µM) for 6 h. The C131A mutation of USP36 impaired its ubiquitination activity and reduced the ability of USP36 to inhibit total YAP poly-ubiquitination in HEK293 cells. **G** USP36 associated with YAP and inhibited YAP K48-linked ubiquitination and degradation in ESCC cells, which promoted the activation of the Hippo/YAP axis and ESCC cancer progression.

Since USP36 belongs to the deubiquitinase family, we subsequently assessed the role of USP36 in YAP ubiquitination. The ubiquitination assay indicated that USP36 depletion could significantly increase the total YAP ubiquitination level, but USP36 overexpression could decrease the total YAP ubiquitination level (Fig. 6A, B). In addition, the ubiquitination assay showed that USP36 overexpression markedly decreased K48-linked ubiquitination but had little effect on K63-linked ubiquitination (Fig. 6C, D). In addition, the ubiquitination assay showed that the USP domain was necessary and sufficient for USP36 to exert its function on YAP polyubiquitination (Fig. 6E). Further ubiquitination assays showed that C131A mutation variants could not inhibit YAP polyubiquitination as the USP36 WT form did (Fig. 6F).

## Discussion

In the present study, we discovered that USP36 is a critical regulator of Hippo signaling in ESCC. We found that USP36 expression was elevated in human esophageal cancer and positively correlated with YAP protein levels in human samples. USP36 interacts with YAP and inhibits YAP polyubiquitination and breakdown, which further promotes esophageal cancer progression (Fig. 6G). Based on these conclusions, we propose that USP36 is a critical effector determining Hippo/YAP status in ESCC, while targeting USP36 protein or modulating its protein level could be a promising strategy in ESCC patients.

According to recent world cancer statistics, there are approximately 570,000 newly diagnosed esophageal cancer cases each year worldwide, and half of them are found in China [1, 30]. With the application of endoscopy, an increasing number of esophageal cancers can be detected early [31]. However, the 5-year overall survival rate of esophageal cancer is still less than 20%, which poses a great challenge [32, 33]. With the progression of molecular biology studies in esophageal cancer [34–36], several novel strategies, such as immunotherapy [37, 38], have been utilized in esophageal cancer treatment. However, there is still a lack of a well-recognized molecular classification system in esophageal cancer, making it difficult to apply personalized therapy. With the application of genomic-based screening in esophageal cancer, several genomic abnormities were observed in ESCC, most of which were clustered in Hippo signaling, Notch signaling, PI3K signaling, and cell cycle signaling [5]. Interestingly, abnormalities in the Hippo pathway, such as FATs mutations, LATS mutations, and YAP amplifications, have been found in 48% of all ESCC patients. Coupled with the molecular biology publications showing the logical link between Hippo signaling and ESCC progression, we posited that the Hippo/YAP axis could be a major driving pathway for ESCC, so understanding YAP protein regulation is of great importance for ESCC therapeutics.

The activation of YAP protein could lead to its translocation into the nucleus, where it interacts with several transcription factors, including TEADs [15, 39]. Targeting the transcriptional function of YAP could be an effective strategy. However, structural biology studies have demonstrated the extensive interaction between YAP and TEADs [40, 41], indicating that such a strategy would be difficult to execute. Thus, interfering with YAP protein stability could be another candidate way to block the Hippo/YAP axis. Since YAP can be polyubiquitinated by several E3 ubiquitin ligases, the ubiquitination level of YAP is critical in the regulation of the Hippo/YAP axis and ESCC progression [7]. Inhibition of certain deubiquitinases, which changes the balance between ubiquitin ligases and deubiquitinases, could turn off the Hippo/YAP axis and inhibit ESCC growth. Actually, several other studies showed a group of deubiquitinases could modulate YAP/TAZ function and tumor progression. Respectively, USP10 and OTUB2 were recently reported as the candidate proteins that deubiquitinated and stabilized YAP/TAZ in the liver and breast cancer models [42, 43]. One study showed that USP14 contributed to YAP/TAZ transcriptional activity and stabilized TAZ but not YAP. Mechanistically, USP14 catalyzed the K48-linked deubiquitination of TAZ to promote TAZ stabilization [44]. Additionally, evidence showed that USP1 functions as a deubiquitinase to regulate TAZ specifically in breast cancer [45]. Our deubiquitinase siRNA screening data identified USP36 as a critical deubiquitinase in modulating YAP stability and ESCC progression. USP36 was first identified in human ovary cancer [29]. Subsequent studies showed that USP36 is located in the nucleus and enhances myc protein stability and myc function. In addition, USP36 was also found to modulate histone function and DNA replication [46]. Thus, USP36 may function as an oncogenic protein in human malignancies. However, our screening data indicated that USP36 is a novel deubiquitinase modulating Hippo signaling function in ESCC. Considering this finding and the other oncogenic role of USP36 in previous studies, blocking USP36 could result in the synchronous inhibition of multiple oncogenic pathways to treat esophageal cancer.

In conclusion, we discovered a novel biological link between the Hippo/YAP axis and USP36 in esophageal carcinoma. Our ESCC human samples and biological studies showed that USP36 was a putative oncogene in promoting ESCC proliferation and metastasis in vitro and in vivo. USP36 could associate with YAP and deubiquitinate YAP protein, which further leads to enhanced Hippo/YAP activity. Based on these findings, regulation of USP36 expression or function could be a promising strategy to treat ESCC.

## Supplementary information

Supplementary Figure legend

Author Contribution Statement

Original films for western blot

Supplementary figure 1

Supplementary figure 2

Supplementary figure 3

Supplementary information

cell line authentication

Language certificate

Ethnic approvement

Data sheet checklist

## Data Availability

The publicly available data are provided in the GEO database. The original siRNA screening data are provided in the supplementary materials. The original data for WB and qPCR are provided in the supplementary materials. The cell line authentication results are shown in the supplementary materials. The ethical approval for the use of human samples is shown in the supplementary materials.
