# Supplementary material for: USP36 facilitates esophageal squamous carcinoma progression via stabilizing YAP
Source: Cell Death Dis. 2022 Dec 5;13(12):1021. doi: 10.1038/s41419-022-05474-5 (PMC9722938; doi:10.1038/s41419-022-05474-5)
Supplement: Supplementary file 1 — Supplementary Figure legend [file 41419_2022_5474_MOESM1_ESM.docx]

**Supplementary Figure 1.** **USP36 was an important regulator in Hippo signaling function in ESCC.**

(**A**) USP36 antibody was verified for IHC by immunofluorescence. Right panel showed quantitative analysis of Integrated density. The Integrated density was markedly decreased in siUSP36 ECA109 cells compared with siControl. (**B**) qPCR results showed that USP36 depletion decreased genes expression of Figure 1I and J in ECA109. (**C**) Western blot showed the total amount of USP36 protein in tumor vs matched normal tissues from ESCC patients. (**D**) Volcano plot of RNA-seq showed that Hippo pathway-related genes were enriched in downregulated genes in ECA109 cells treated with siUSP36. Threshold P<0.05 and |log2fold change| >1 were set as screening criteria. (**E, F**) Top 20 GO-BP terms and KEGG pathways enriched in differentially expressed genes in RNA-seq data of USP36 siRNA ECA109 cells. (**G**) The quantification of the positive correlation between USP36 and the YAP and TAZ target genes.

**Supplementary Figure 2.** **USP36 regulates Hippo/YAP axis in human ESCC cells.**

(**A**) USP36 silencing didn’t affect the expression levels of upstream components of the Hippo pathway. Western blot showed protein expression of MST1, MST2, LAST1, LATS2, p-YAP(ser127), ACTB. (**B**) Western blot showed protein expression of p-YAP(ser127) and YAP. Right panel showed the ratio of p-YAP(ser127) over total YAP signal and they should not see any difference in siUSP36 vs sicontrol cells. (**C**) Cell viability was determined by CCK8 assay in ESCC cell lines transfected with siControl or two independent USP36 siRNA. CCK-8 assay showed that USP36 depletion inhibited the proliferation of EC9706cells. (**D**) EdU assay to show the cell proliferation of ESCC cell lines transfected with siControl or two independent USP36 siRNA. EC9706 cells were labeled with EdU. Right panel showed quantification of EdU results. Green denoted EdU-positive cells; blue denoted cell nuclei; scale bar, 250 µm. (**E**) Transwell assay of EC9706 cells transfected with siControl or two independent USP36 siRNA, respectively. Depletion of endogenous USP36 markedly decreased cell migration. Right panel showed quantification of transwell assay results. Scale bar, 250 µm. (**F**) Wound healing assay of ESCC cell lines migration capability following transfected with siControl or two independent USP36 siRNA. The migration assay showed that depletion of USP36 suppressed the migration of EC9706 cells. Scale bar, 250 µm. Right panel showed quantification of wound healing results. (**G**) Colony formation of EC9706 cells transfected with siControl or two independent USP36 siRNA, respectively, showed that USP36 depletion inhibited cell proliferation. Right panel showed quantification of colony formation assay results. Scale bar, 250 µm. (**H**) Representative plots (left panel) of apoptosis EC9706 cells transfected with siControl or two independent USP36 siRNA, respectively. USP36 depletion promoted the apoptosis of EC9706 cells. Quantitative summary (right panel) of apoptosis analysis of FACS. (**I**) The expression of the Caspase3 (cleaved) protein was detected by western blotting. USP36 depletion promoted the apoptosis of ECA109, KYSE150 and EC9706 cells. (**J**) 3D culture assay of ECA109, KYSE150 and EC9706 cells transfected with siControl or two independent USP36 siRNA, respectively, showed that USP36 depletion inhibited anchor-independent growth ability. Right panel showed quantification of 3D culture assay results. Scale bar, 100 µm. (**K**) Representative image of ESCC tumor growth in vivo. **P<0.01, ***P<0.001, ****P<0.0001.

**Supplementary Figure 3.** **USP36 was required for ESCC cell progression through Hippo/YAP signaling.**

(**A**) The expression of the Caspase3 (cleaved) protein was detected by western blotting. Overexpression of USP36 inhibited apoptosis in ECA109 cells. (**B**) ECA109 cells were transduced with lentiviruses expressing vector or USP36-Flag. 3D culture assays showed that overexpression of USP36 activated proliferation and anchor-independent growth in ECA109 cells. Right panel showed quantification of 3D culture assay results. Scale bar, 100 µm. (**C**) Representative image of ESCC tumor growth in vivo. (**D**-**E**) qPCR analysis of USP36 or YAP mRNA levels to validate knockdown or overexpression efficiency in KYSE150 cells. (**F-M**) KYSE150 cells were transduced with lentiviruses expressing control or shRNA targeting USP36, following with YAP overexpression. (**F**) USP36 depletion decreased Hippo target gene expression, which was rescued by YAP overexpression in KYSE150 cells. (**G**) USP36 depletion inhibited TEAD response element activity in KYSE150 cells, and this effect could be attenuated by YAP overexpression. (**H**) EdU assays showed that USP36 depletion inhibited the proliferative ability of KYSE150 cells, which could be rescued by YAP overexpression in KYSE150 cells. KYSE150 cells were labeled with EdU. Right panel showed quantification of EdU results. Green denoted EdU-positive cells; blue denoted cell nuclei; scale bar, 250 µm. (**I**) Transwell assays showed that USP36 depletion decreased cell migration and invasion, which could be rescued by YAP overexpression in KYSE150 cells. Right panel showed quantification of transwell assay results. Scale bar, 250 µm. (**J**) The expression of the Caspase3(cleaved) protein was detected by western blotting. USP36 depletion promoted the apoptosis of KYSE150 cells. (**K**) 3D culture assay showed that USP36 depletion inhibited anchor-independent growth ability, which could be rescued by YAP overexpression in KYSE150 cells. Right panel showed quantification of 3D culture assay results. Scale bar, 100 µm. (**L**) Depletion of USP36 markedly decreased cell migration, as shown by a wound healing assay, and this effect could be attenuated by YAP overexpression in KYSE150 cells. Scale bar, 250 µm. (**M**) Representative image of ESCC tumor growth in vivo. (**N**) Western blot of the distribution of YAP in nucleo-cytoplasmic extracts. *P<0.05, **P<0.01, ***P<0.001.
