# Supplementary material for: USP36 facilitates esophageal squamous carcinoma progression via stabilizing YAP
Source: Cell Death Dis. 2022 Dec 5;13(12):1021. doi: 10.1038/s41419-022-05474-5 (PMC9722938; doi:10.1038/s41419-022-05474-5)
Supplement: Supplementary file 2 — Author Contribution Statement [file 41419_2022_5474_MOESM2_ESM.docx]

**Author Contribution Statement：**Wenhao Zhang, Junwen Luo, Zhaohua Xiao performed most of the bench work. Yifeng Zang performed the screening assay. Xin Li, Youjia Zhou, Jie Zhou performed the IHC assay and data analysis. Zhongxian Tian, Jian Zhu and Xiaogang Zhao supervised the process of the study and performed the manuscript writing. All authors read and approve the final manuscript.
