# Supplementary material for: USP36 facilitates esophageal squamous carcinoma progression via stabilizing YAP
Source: Cell Death Dis. 2022 Dec 5;13(12):1021. doi: 10.1038/s41419-022-05474-5 (PMC9722938; doi:10.1038/s41419-022-05474-5)

Figure.2B

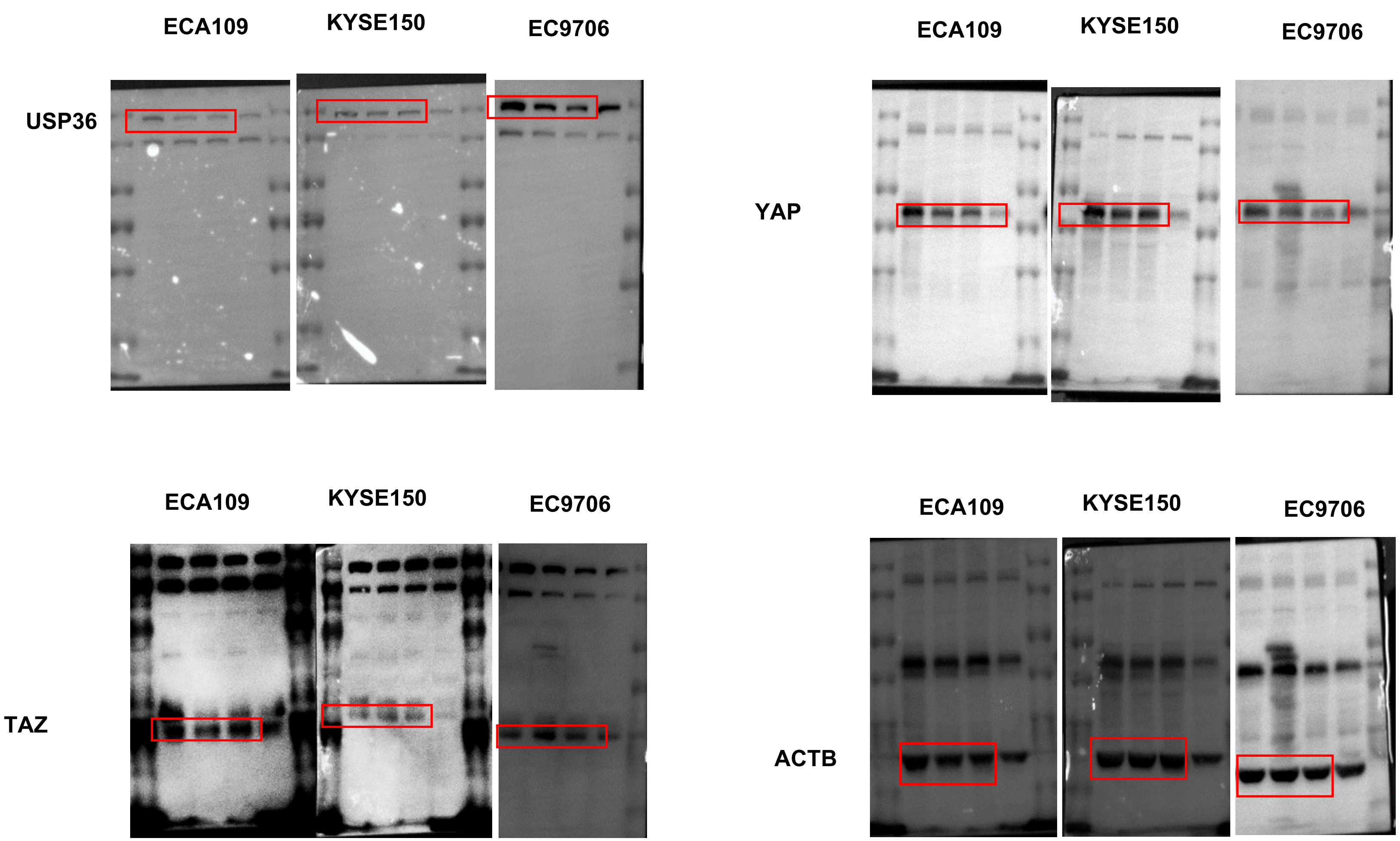

ECA109
KYSE150
EC9706
USP36
ECA109
KYSE150
EC9706
YAP
ECA109
KYSE150
EC9706
TAZ
ECA109
KYSE150
EC9706
ACTB

Figure.2F

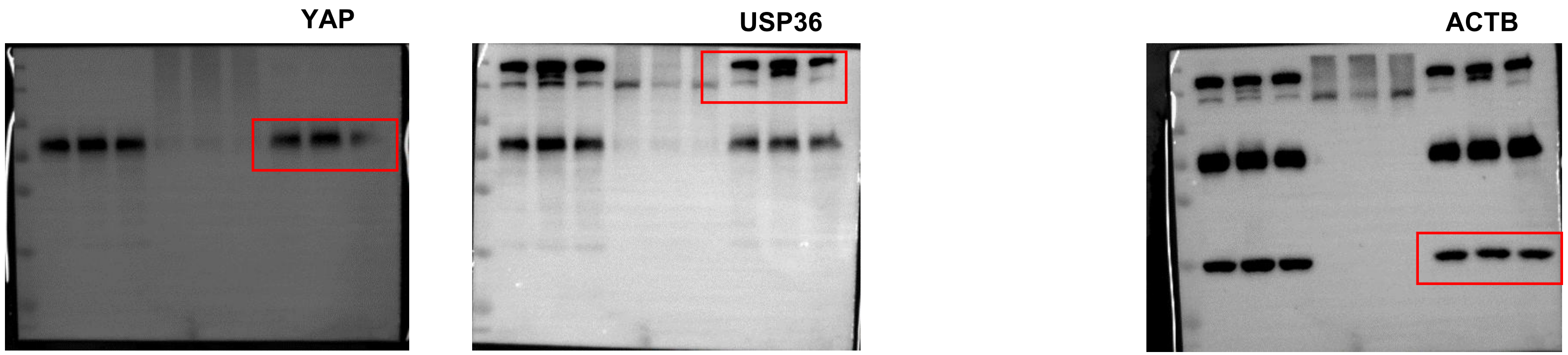

Figure.4J

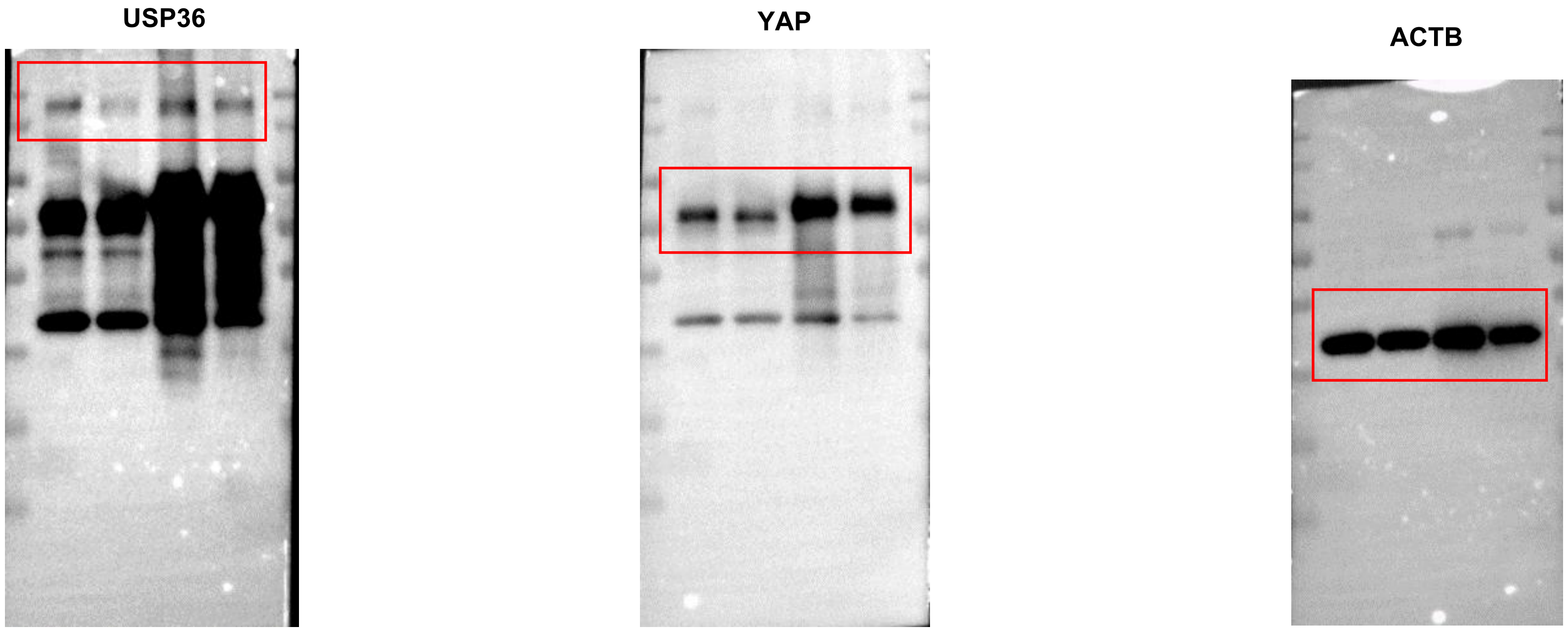

Figure.5B

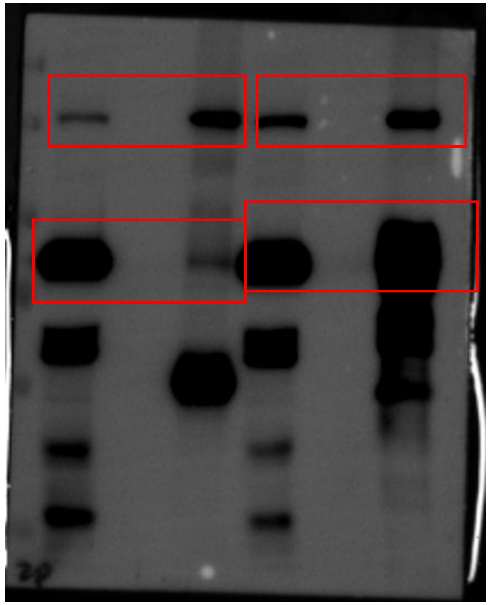

Figure.5C

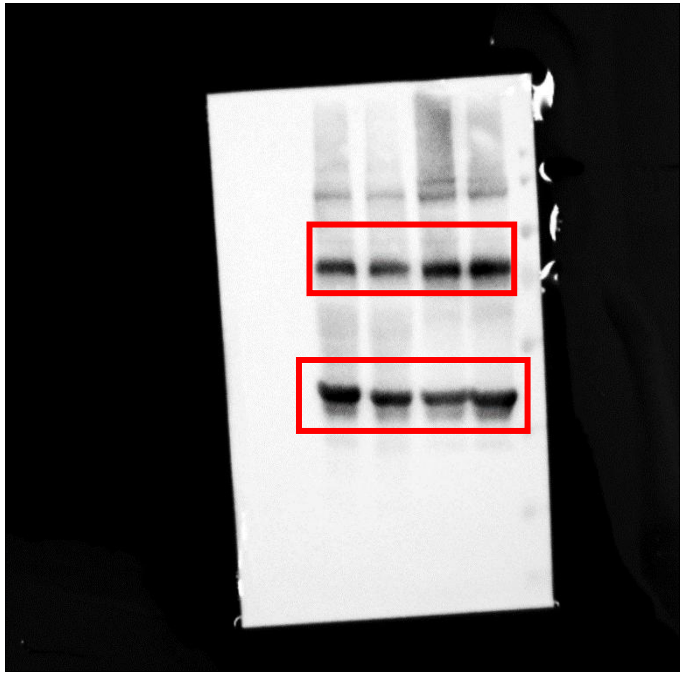

Figure.5D

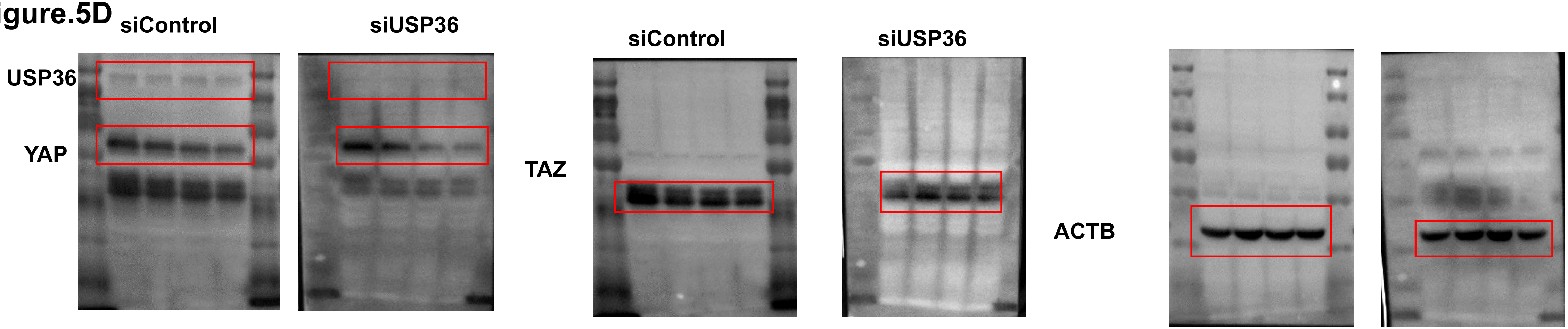

Figure.5E

Input-Myc

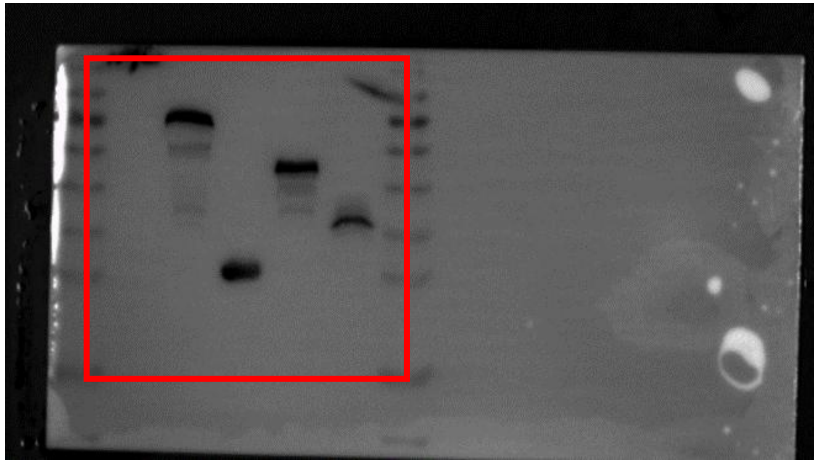

Input-Flag

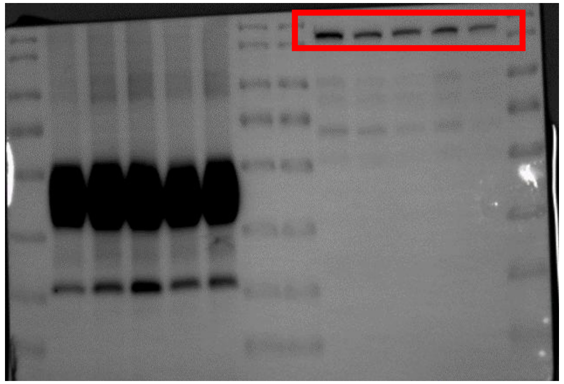

Input-ACTB

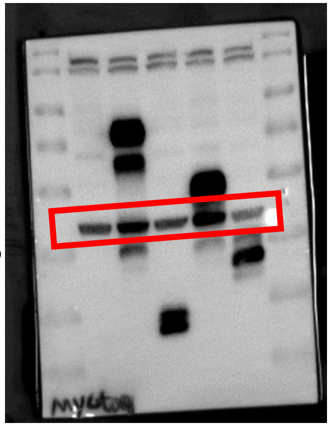

IP-Flag

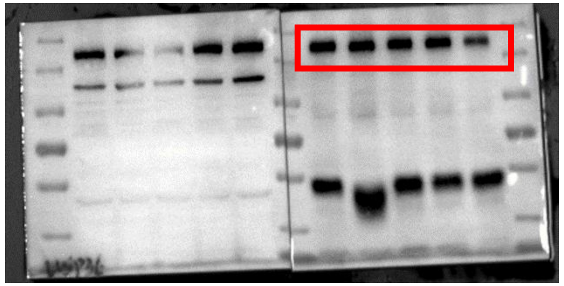

IP-Myc

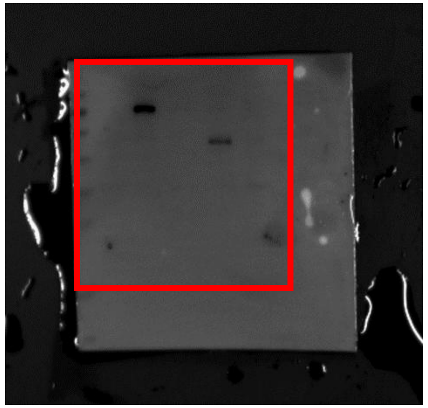

Figure.5F

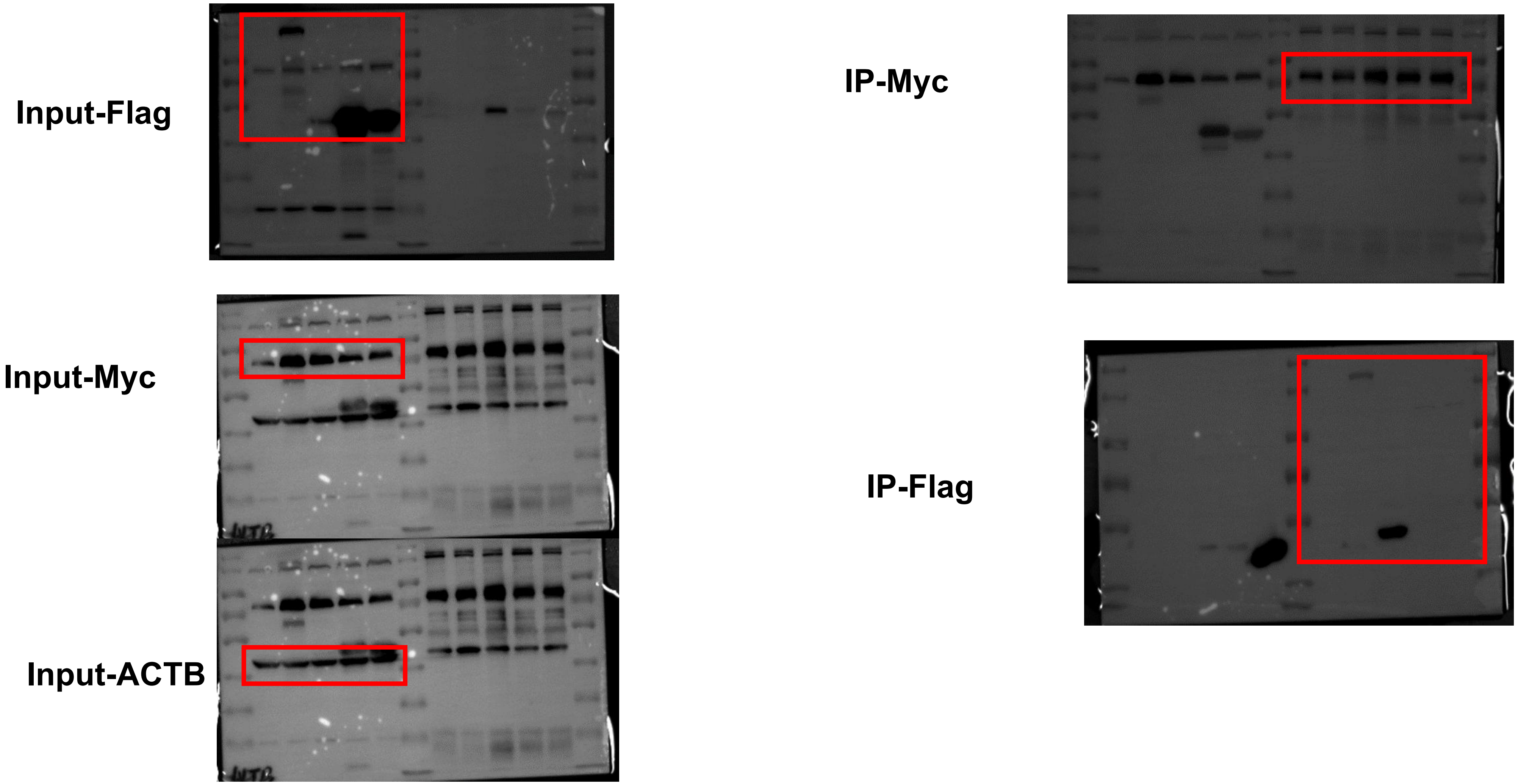

Figure.5G

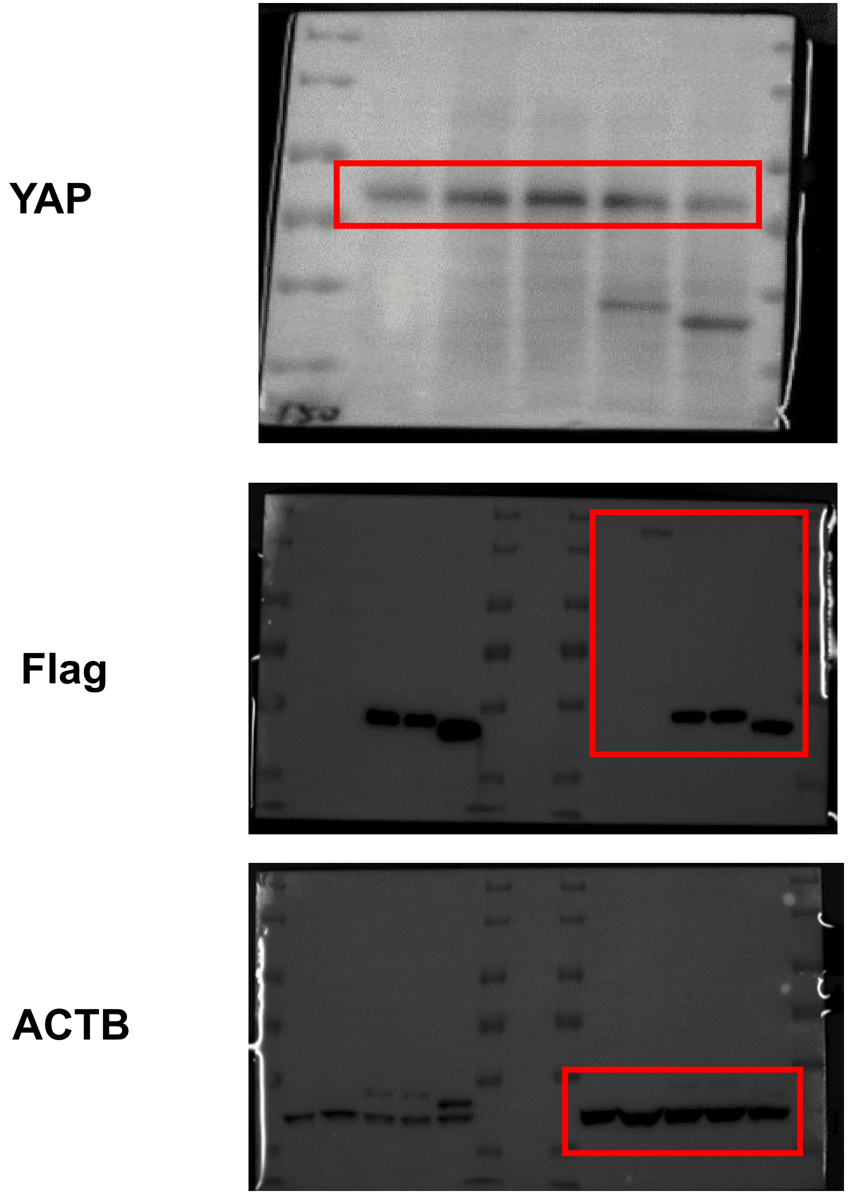

Figure.5I

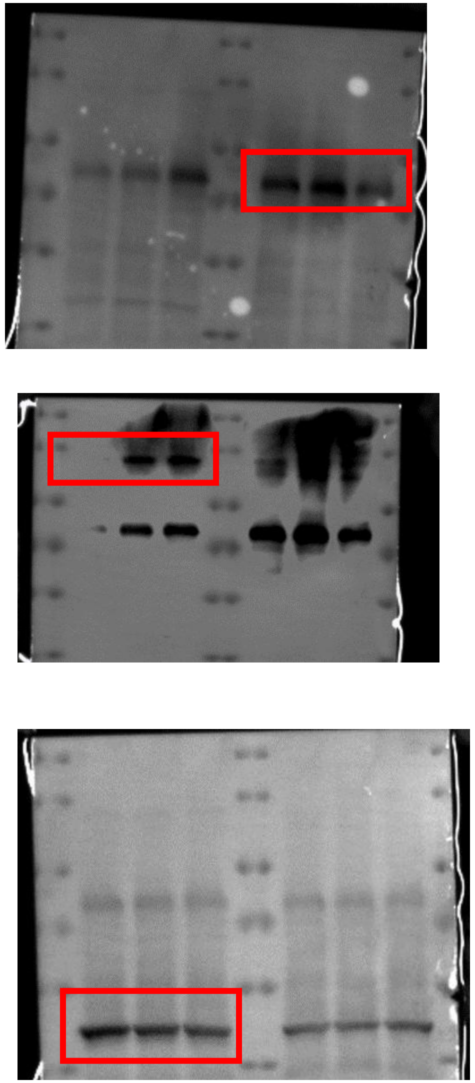

Figure.6A

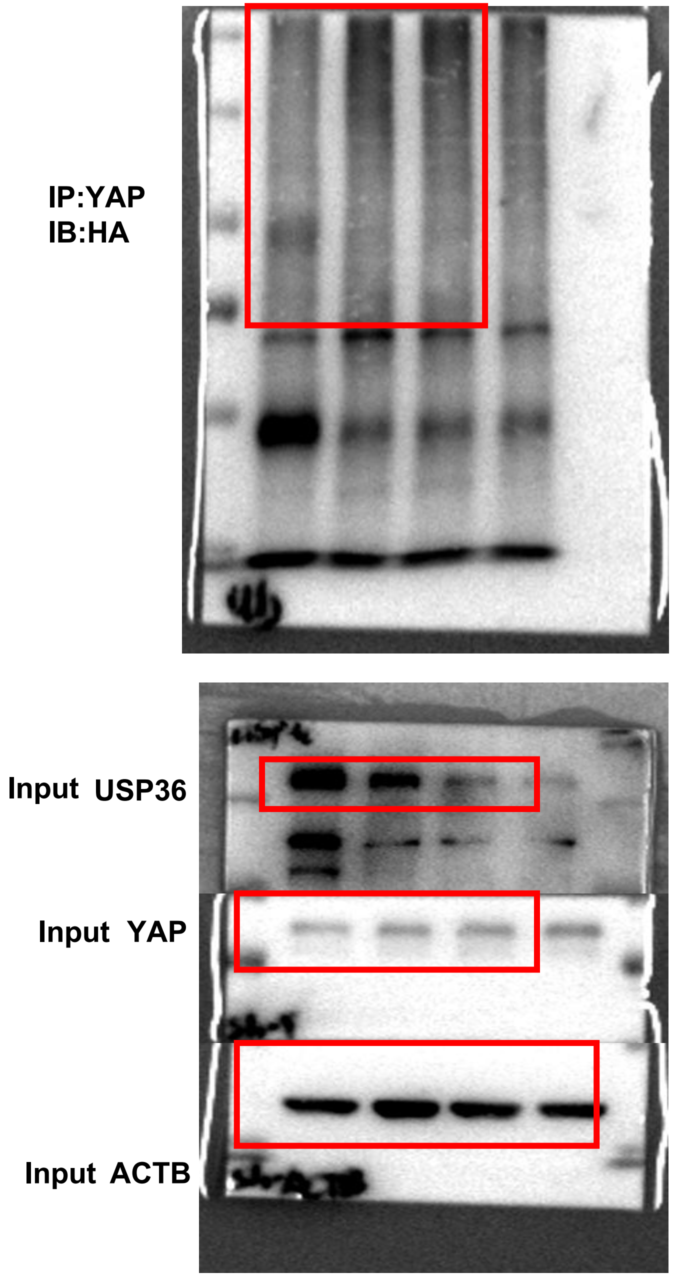

Figure.6B,C,D

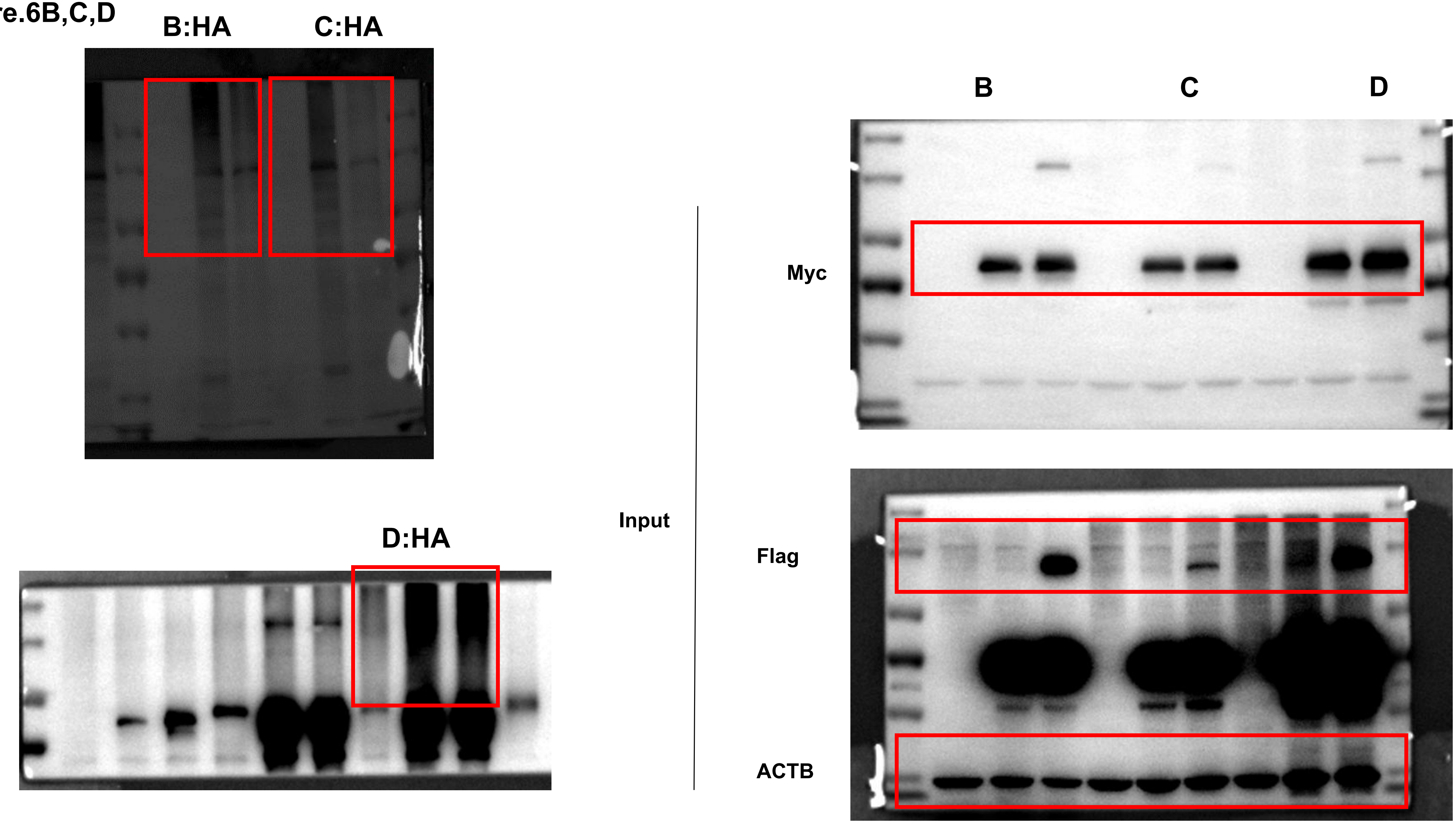

Figure.6E,F

E:HA

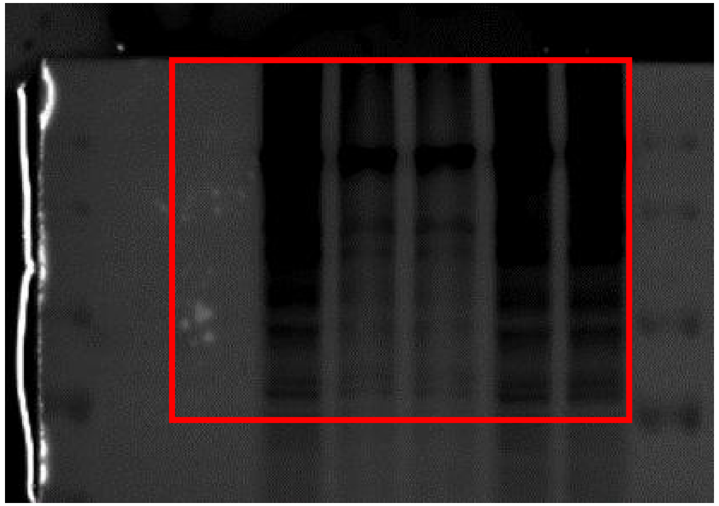

E:FLAG

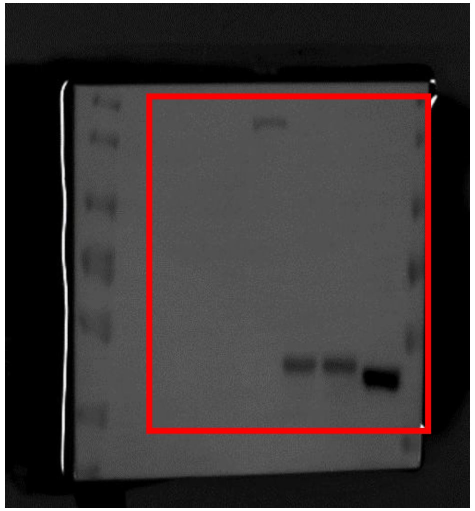

F:HA

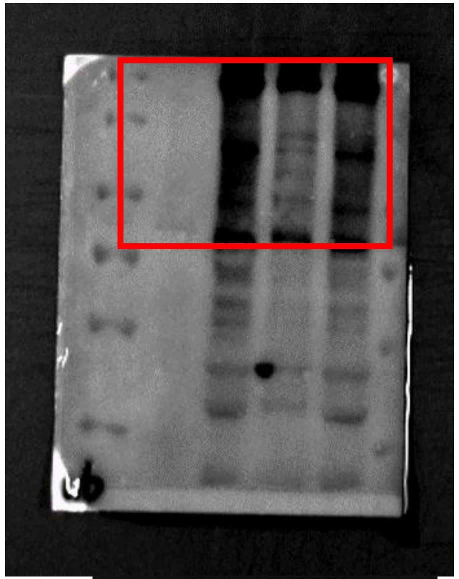

E:MYC

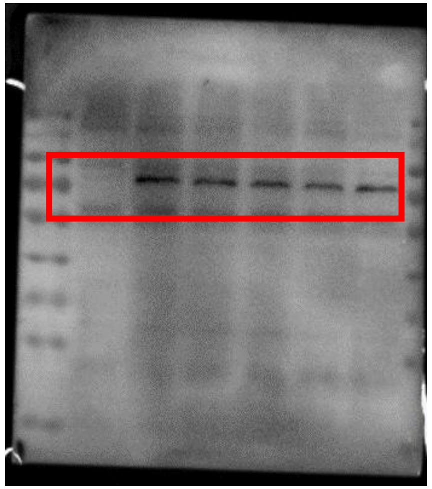

E:ACTB

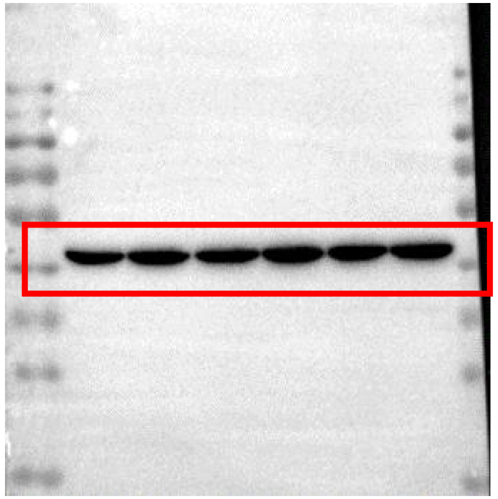

F:FLAG

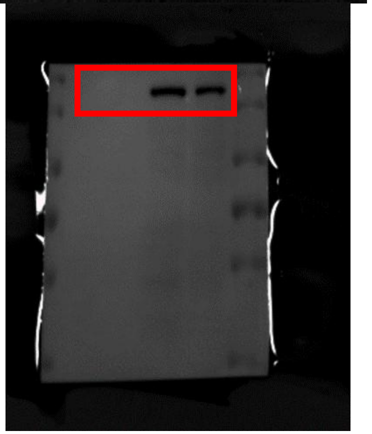

Figure.S1C

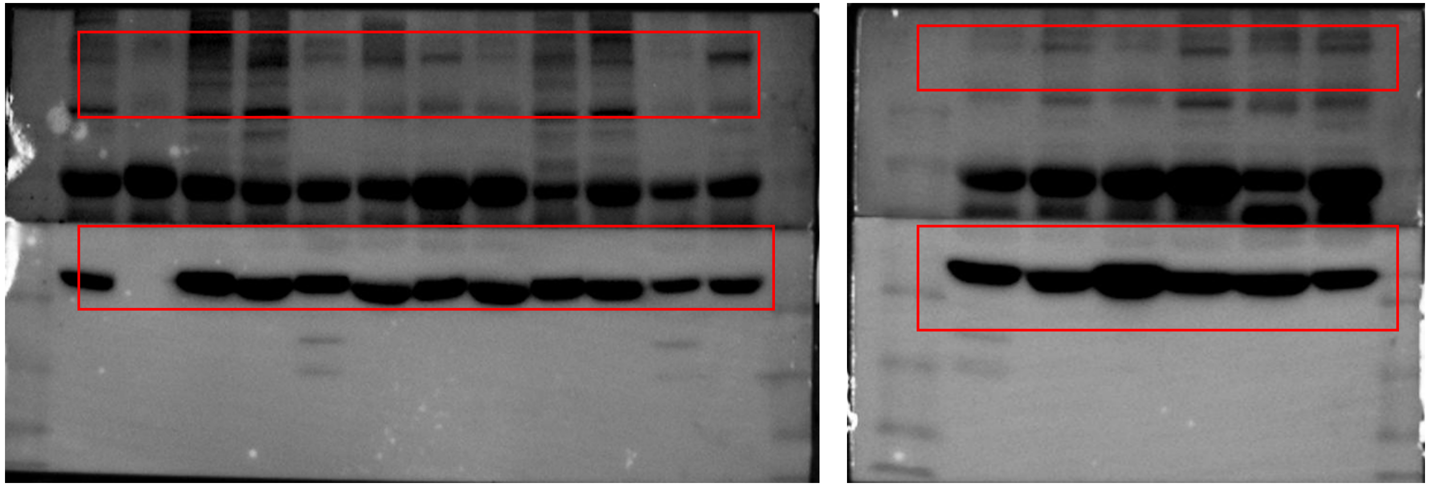

Figure.S2A

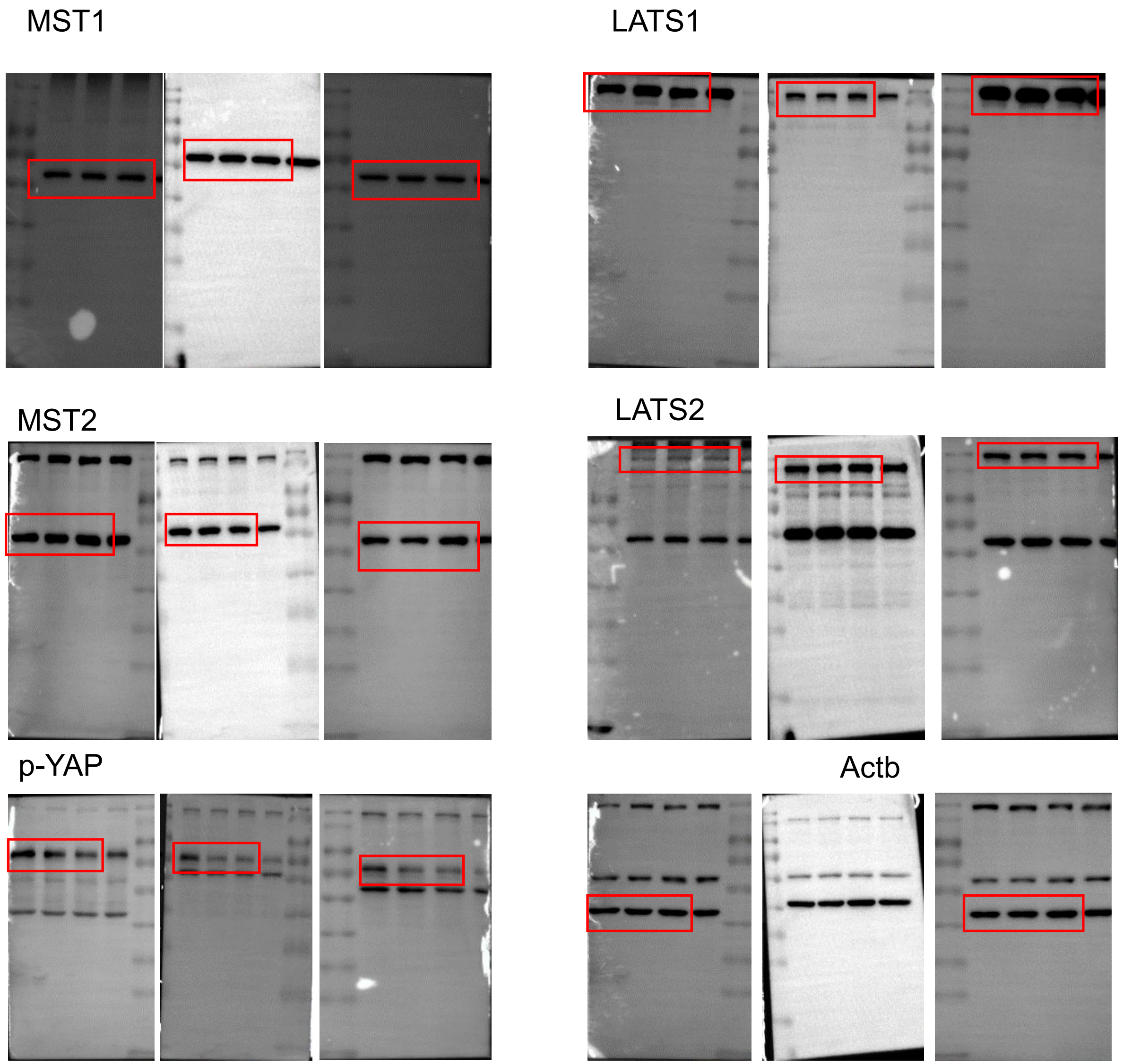

Figure.S2B

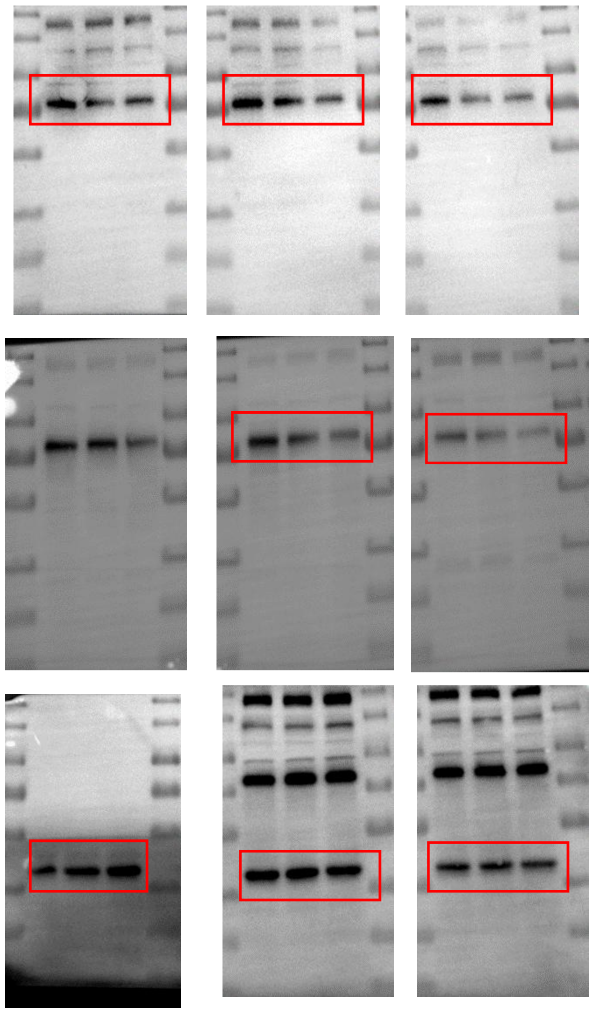

Figure.S2H

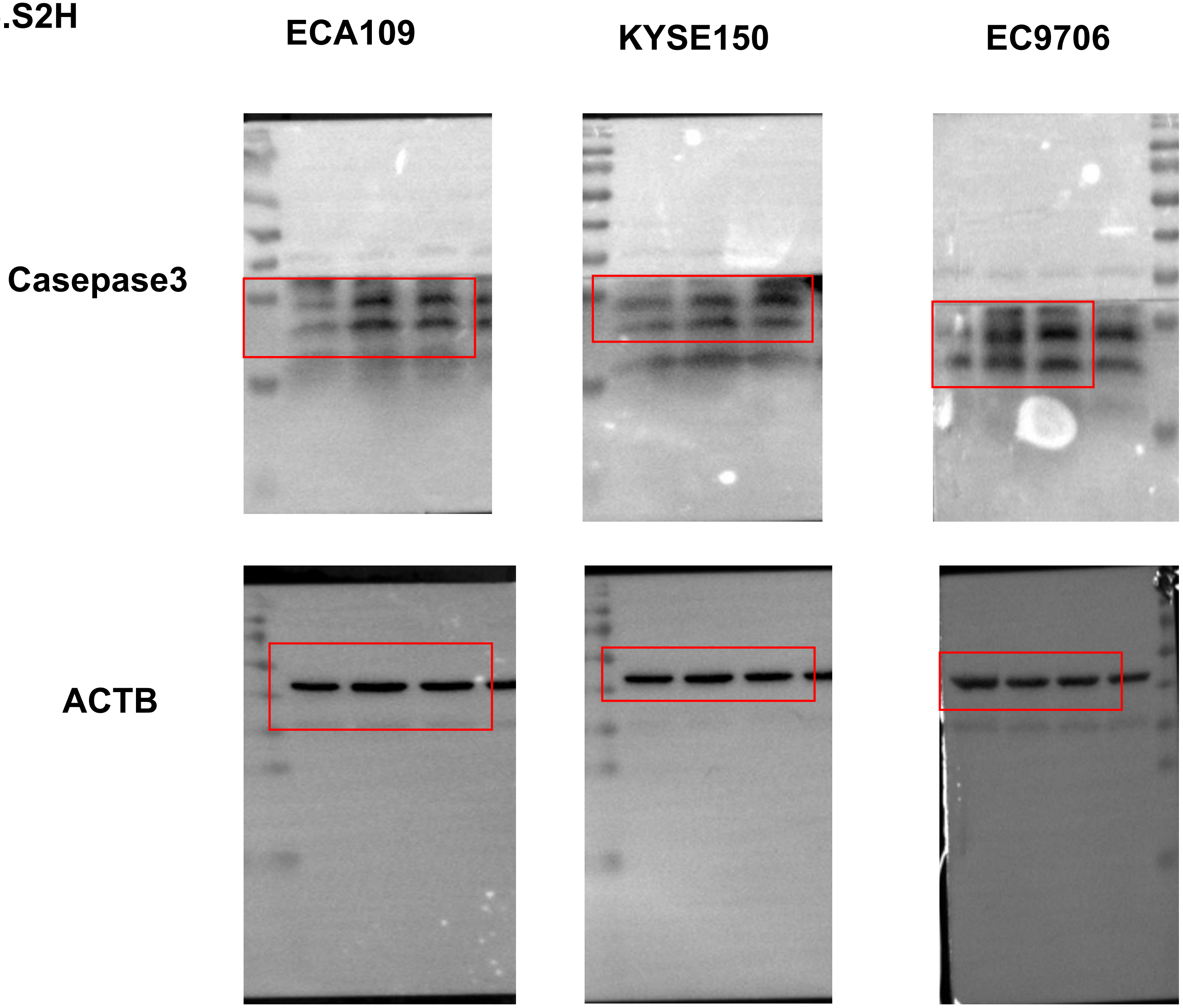

Figure.S3A

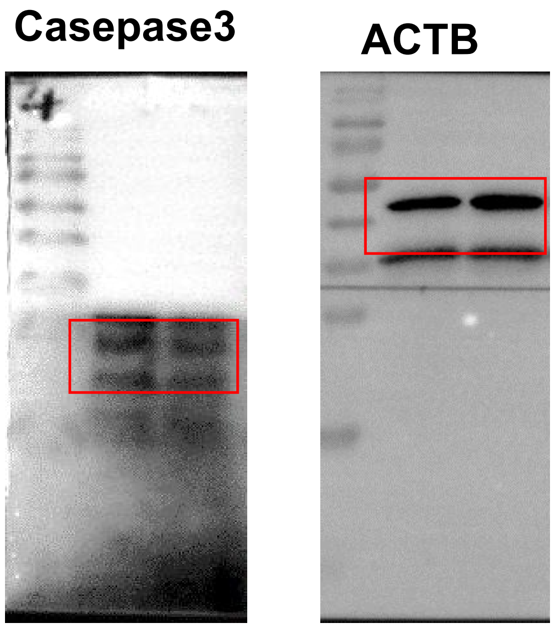

Figure.S3J

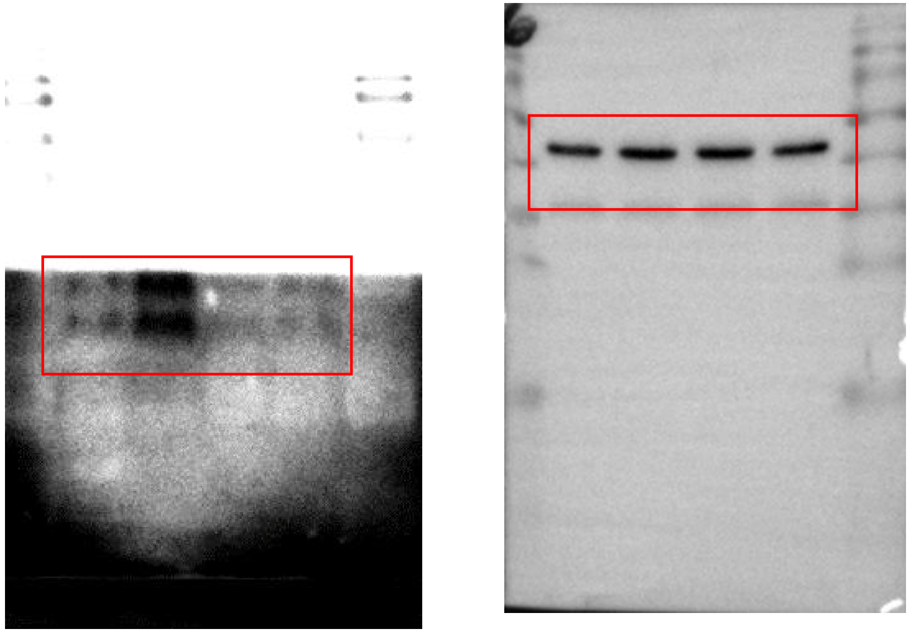

Figure.S3N

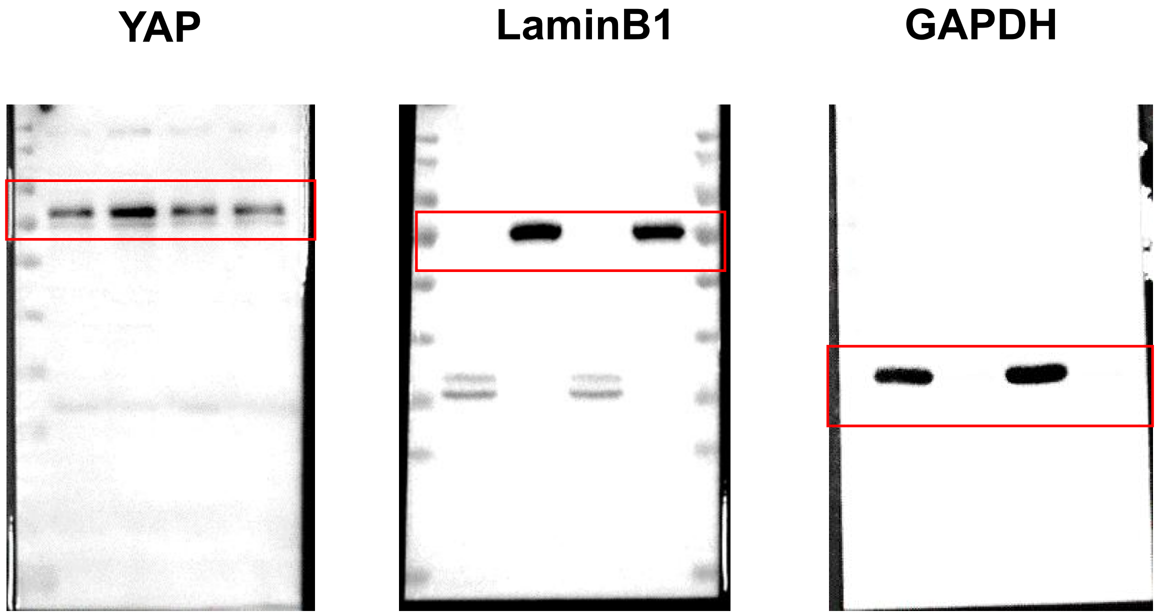

Supplement: Supplementary file 3 — Original films for western blot [file 41419_2022_5474_MOESM3_ESM.pdf]
