# Supplementary figures and images for: USP36 facilitates esophageal squamous carcinoma progression via stabilizing YAP
Source: Cell Death Dis. 2022 Dec 5;13(12):1021. doi: 10.1038/s41419-022-05474-5 (PMC9722938; doi:10.1038/s41419-022-05474-5)

Figure. S1

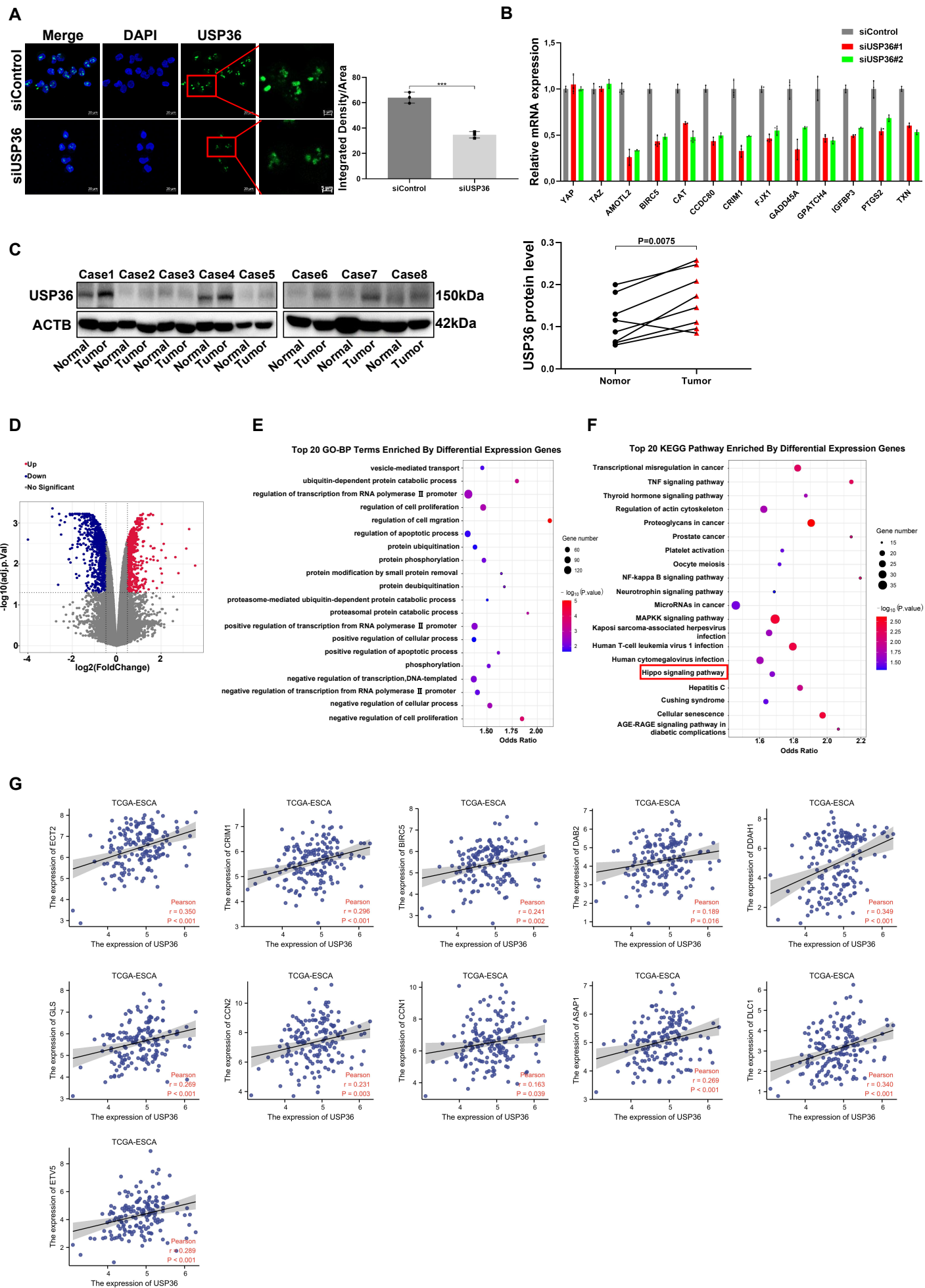

Supplement: Supplementary file 4 — Supplementary figure 1 [file 41419_2022_5474_MOESM4_ESM.pdf]

Figure. S2

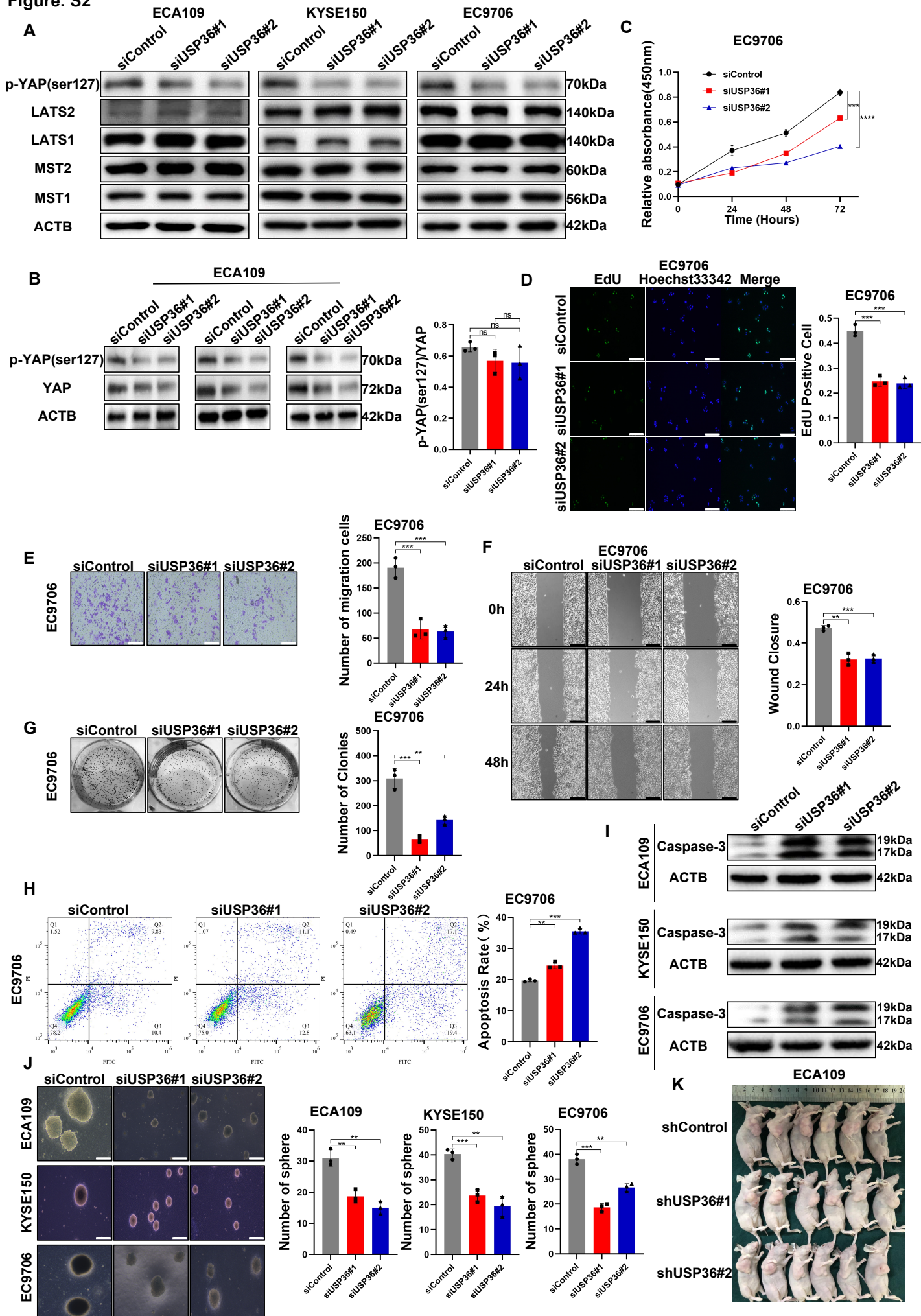

Supplement: Supplementary file 5 — Supplementary figure 2 [file 41419_2022_5474_MOESM5_ESM.pdf]

Figure. S3

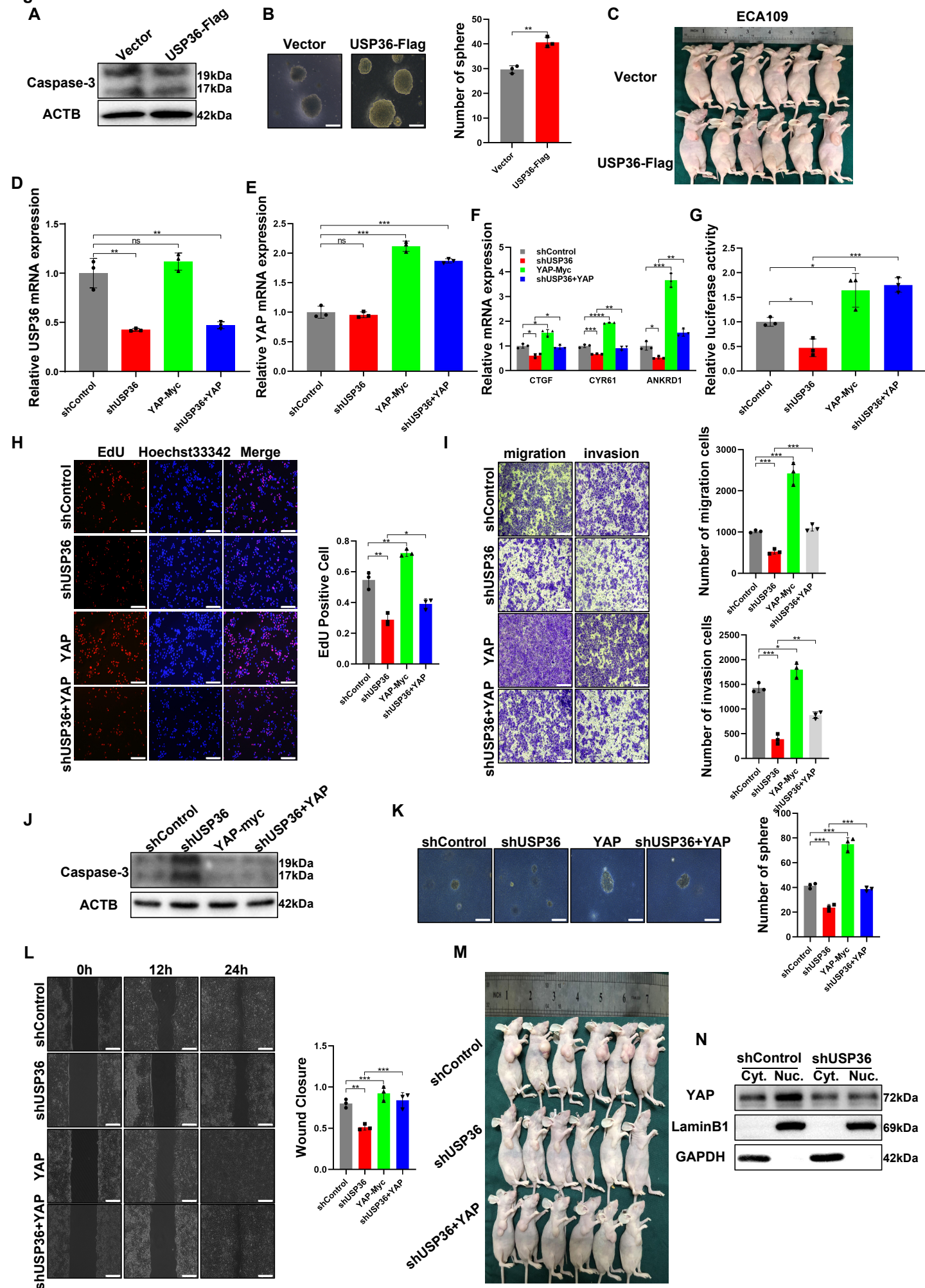

Supplement: Supplementary file 6 — Supplementary figure 3 [file 41419_2022_5474_MOESM6_ESM.pdf]
