# Supplementary material for: USP36 facilitates esophageal squamous carcinoma progression via stabilizing YAP
Source: Cell Death Dis. 2022 Dec 5;13(12):1021. doi: 10.1038/s41419-022-05474-5 (PMC9722938; doi:10.1038/s41419-022-05474-5)
Supplement: Supplementary file 7 — Supplementary information [file 41419_2022_5474_MOESM7_ESM.docx]

**Supplemental information**

**Supplementary Table 1.** Sequences of siUSP36,siYAP,shUSP36

| **Gene** | **Sequences** |
| --- | --- |
| siUSP36#1 | 5’-GCAAAUAUGUGUUGCUCAATT-3’  5’-UUGAGCAACACAUAUUUGCTT-3’ |
| siUSP36#2 | 5’-CCGGCAAGCUGCGAAUAUUTT-3’  5’-AAUAUUCGCAGCUUGCCGGTT-3’ |
| siControl | 5’-UUCUCCGAACGUGUCACGUTT-3’  5’-ACGUGACACGUUCGGAGAATT-3’ |
| siYAP | 5’-GGUCAGAGAUACUUCUUAAAU-3’  5’-UUAAGAAGUAUCUCUGACCAG-3’ |
| shUSP36#1 | F: 5’-GATCGATCCGGCAAGCTGCGAATATTTCAAGACAATATTCGCAGCTTGCCGGATCTTTTT-3’  R: 5’-CGCGAAAAAGATCCGGCAAGCTGCGAATATTGTCTTGAAATATTCGCAGCTTGCCGGATC-3’ |
| shUSP36#2 | F: 5’-GATCGACTACCTTGGTCCATCAAATTTTCAAGACAAATTTGATGGACCAAGGTAGTTTTTT-3’  R: CGCGAAAAAACTACCTTGGTCCATCAAATTTGTCTTGAAAATTTGATGGACCAAGGTAGTC-3’ |

**Supplementary Table 2.** Primer sequences for qRT-PCR

| **Gene** | **Primer Sequences** |
| --- | --- |
| CTGF | F: 5’-CGCACAAGGGCCTATTCTGT-3’  R: 5’-GAGCACCATCTTTGGCGGT-3’ |
| CYR61 | F: 5’-AGCAGCCTGAAAAAGGGCAA-3’  R: 5’-AGCCTGTAGAAGGGAAACGC-3’ |
| ANKRD1 | F: 5’-AGACACTTCTAGCCCACCCT-3’  R: 5’-AGCTCTGCCTCTCGTTGTTT-3’ |
| USP36 | F: 5’-TTCGGCACAGCTGCTCTC-3’  R: 5’-CTCGGGGACAACAGCATCTT-3’ |
| 36B4 | F: 5’-CGTCCTCGTGGAAGTGACAT-3’  R: 5’-GCATCATGGTGTTCTTGCCC-3’ |
| AMOTL2 | F:5’-GGGTGATTCAGTTGGGTGCT-3’  R:5’-CGAGGAGTCTTCCAGTGTCC-3’ |
| BIRC5 | F:5’- ATGACGACCCCATAGAGGAAC-3’  R:5’-CGCACTTTCTCCGCAGTTTC-3’ |
| CAT | F:5’-CTCCGGAACAACAGCCTTCT-3’  R:5’-ATAGAATGCCCGCACCTGAG-3’ |
| CCDC80 | F:5’-GATCTGTGGCGGTACTCTGA-3’  R:5’-CATTGTGTAATCCAATGGTGGC-3’ |
| CRIM1 | F:5’-GGCGTTTGCGAAGATGAGAA-3’  R:5’-TTGCTGCAGGTTCGAATGGT-3’ |
| FJX1 | F:5’- AACTTGCGTTTGAGCCGTTG-3’  R:5’-CCCCTGCTTGGAACAGTGAT-3’ |
| GADD45A | F:5’-AAGGATGGATAAGGTGGGGGA-3’  R:5’-CACGTTATCGGGGTCGACGTT-3’ |
| GPATCH4 | F:5’-CCCAGCAATGACGAGGTCAA-3’  R:5’-ACCCTGAGAGCCTGAGTGAT-3’ |
| IGFBP3 | F:5’-GTGCGGCATCTACACCGAG-3’  R:5’-CTCACTAGCATTTCCTGGCG-3’ |
| PTGS2 | F:5’-TCCCTTGGGTGTCAAAGGTAAA-3’  R:5’-TGGCCCTCGCTTATGATCTG-3’ |
| TXN | F:5’-ATGTGGATGACTGTCAGGATGT-3’  R:5’-GGTGGCTTCAAGCTTTTCCT-3’ |

**Abbreviations:** F, forward; R, reverse.

**Supplementary Table 3.** Antibodies used in the present study.

| **Gene** | **Source** | **No. of Catalogue** |
| --- | --- | --- |
| Anti-USP36(R) | Proteintech | 14783-1-AP |
| Anti-USP36(M) | Proteintech | 68165-1-Ig |
| Anti-YAP1(R) | Proteintech | 13584-1-AP |
| Anti-YAP1(M) | Proteintech | 66900-1-lg |
| Anti-MST1 | Proteintech | 22245-1-AP |
| Anti-MST2 | Proteintech | 12097-1-AP |
| Anti-LATS1 | Cell Signaling Technology | 9153S |
| Anti-LATS2 | Cell Signaling Technology | 5888T |
| p-YAP(Ser127) | Cell Signaling Technology | 13008F |
| Anti-Caspase-3 | Cell Signaling Technology | 9662S |
| Anti-Flag-tag | Cell Signaling Technology | 14793S |
| Anti-Myc-tag | Cell Signaling Technology | 2278S |
| Anti-UB | Proteintech | 10201-2-AP |
| Anti-HA-tag | Proteintech | 51064-2-AP |
| Anti-β-actin | Proteintech | 20536-1-AP |
| Rabbit IgG | Beyotime | A7016 |
| Mouse IgG | Beyotime | A7028 |
| Goat Anti-Rabbit IgG | ZSGB-BIO | ZB-2301 |
| Goat Anti-Mouse IgG | ZSGB-BIO | ZB-2305 |
| Goat Anti-Mouse IgG H&L(Alexa Fluor647) | abcam | ab150115 |
| CoraLite488conjugated Affinipure Goat Anti-Rabbit IgG(H&L) | Proteintech | SA00013-2 |
