# Supplementary material for: USP36 facilitates esophageal squamous carcinoma progression via stabilizing YAP
Source: Cell Death Dis. 2022 Dec 5;13(12):1021. doi: 10.1038/s41419-022-05474-5 (PMC9722938; doi:10.1038/s41419-022-05474-5)
Supplement: Supplementary file 8 — cell line authentication [file 41419_2022_5474_MOESM8_ESM.pdf]

0871-65011065　km-tech@tsingke.net(项目)

细胞鉴定实验报告——STR 鉴定

项目开始日：2020 年 9 月 21 日

项目结束日：2020 年 9 月 24 日

实验人	报告人	审核人
李旭丽	李旭丽	李宝燕

样品信息

1. 样品名称

KYSE-150

2 样品类型

细胞沉淀

实验方法

1. 提取细胞基因组 DNA

使用 Tsingke 的动物基因组抽提试剂盒（货号 TSP201-200）提取细胞的基因组 DNA。

2. 使用 20 位点特异荧光引物进行扩增

使用 Tsingke 的金牌 Mix(green)（货号 TSE101）进行扩增。

实验结果

样品编号	多等位基因	匹配细胞系	细胞库	EV 值	匹配说明
KYSE-150	无	KYSE-150	CLASTR	1.00	完全匹配

- 多等位基因指三等位及以上基因现象。

备注：待测细胞系与收录于 CLASTR,ATCC, DSMZ, JCRB 和 RIKEN 数据库的细胞系 STR 数据进行比对，未收录于以上细胞库的细胞系将无法匹配。

0871-65011065 km-tech@tsingke.net(项目)

样本分型结果详情

细胞 KYSE-150 的 STR 位点和 Amelogenin 位点的基因分型结果						
	送检细胞 STR 信息			细胞库细胞 STR 信息		
	送检细胞名:KYSE-150			细胞库细胞名: KYSE-150		
Loci	Allele1	Allele2	Allele3	Allele1	Allele2	Allele3
D5S818	12	13		12	13	
D13S317	8	11		8	11	
D7S820	10	11		10	11	
D16S539	9	11		9	11	
VWA	16	17		16	17	
TH01	7	9		7	9	
AMEL	x			X		
TPOX	8			8		
CSF1PO	12	13		12	13	
D12S391	19	22				
FGA	21	24		21	24	
D2S1338	25					
D21S11	30	31		30	31	
D18S51	14			14		
D8S1179	10	15		10	15	
D3S1358	15	16		15	16	
D6S1043	18	20				
PENTAE				12	18	
D19S433	15	15.2				
PENTAD				10		

细胞遗传质量鉴定检测

Cell Line Authentication Service

STR 基因型检测报告

样 品 信 息

样品编号：

客户样本编号	公司编号
EC9706	20180528-04

样品数量： 1

样品性状： 细胞系

检测项目： STR

送检单位： 北纳

检测方法：用 Axygen 的基因组抽提试剂盒提取 DNA，采用 20- STR 扩增方案扩增，在 ABI 3730XL 型遗传分析仪上对 STR 位点和性别基因 Amelogenin 进行检测。

检 测 结 果

(一)　检验基本情况

	多等位基因	匹配细胞系	细胞库	EV 值	匹配说明
20180528-04	无	EC9706	CRC	1.00	完全匹配

样本基因型检验结果

- 多等位基因指三等位及以上基因现象。
- 本次检测各细胞分型结果良好。
- **各样本描述**
- 20180528-04：该株细胞 DNA 分型在细胞系检索中找到**完全匹配**的细胞系，CRC 数据库显示细胞名为 **EC9706**，细胞号对应**69**。本次检测在该细胞系中**没有发现多等位基因**。

  备注：待测细胞系与收录于 ATCC, DSMZ, JCRB 和 RIKEN 数据库的细胞系 STR 数据进行比对，未收录于以上细胞库的细胞系将无法匹配。

样本分型结果

细胞 20180528-04 的 STR 位点和 Amelogenin 位点的基因分型结果						
Loci	送检细胞 STR 信息			细胞库细胞 STR 信息		
	送检细胞名：EC9706			细胞库细胞名：EC9706		
	Allele1	Allele2	Allele3	Allele1	Allele2	Allele3
D5S818	11	12		11	12	

D13S317	12	13.3		12	13.3	
D7S820	8	12		8	12	
D16S539	9	10		9	10	
VWA	16	18		16	18	
TH01	7	7		7	7	
AMEL	X	X		X	X	
TPOX	8	12		8	12	
CSF1PO	9	10		9	10	
D12S391	20	25				
FGA	21	21				
D2S1338	17	17				
D21S11	27	28				
D18S51	16	16				
D8S1179	12	13				
D3S1358	18	18				
D6S1043	18	19				
PENTAE	7	17				
D19S433	13	14				
PENTAD	8	15				

其 他 说 明

(一) 分型方案及位点分布

	方案 1	方案 2	方案 3	方案 4
1	TH01	TPOX	D3S1358	AMEL
2	D12S391	VWA	D13S317	D5S818
3	D7S820	D8S1179	D6S1043	D2S1338
4	CSF1PO	PENTAD	D16S539	D21S11
5	FGA		D19S433	D18S51
6	PENTAE			

实验方案及位点

(二) STR 数据库比对

本公司采用 DSMZ tools 进行细胞系比对，其中包含来自于 ATCC, DSMZ, JCRB 和 RIKEN 数据库的 2455 个细胞系 STR 数据。如果待检测细胞未收录于以上细胞库或这是自行建立的新细胞系将无法进行比对，用户需根据细胞分型结果自行与其他数据库进行比对。

签发日期：2018 年 06 月 04 日

Sample File	Sample Name	Panel	SQO	OS	SQ
44_D06_CellLineAthentication-1-0530.fsa	EC9706	21Plex_STR_Panel_v0.4			

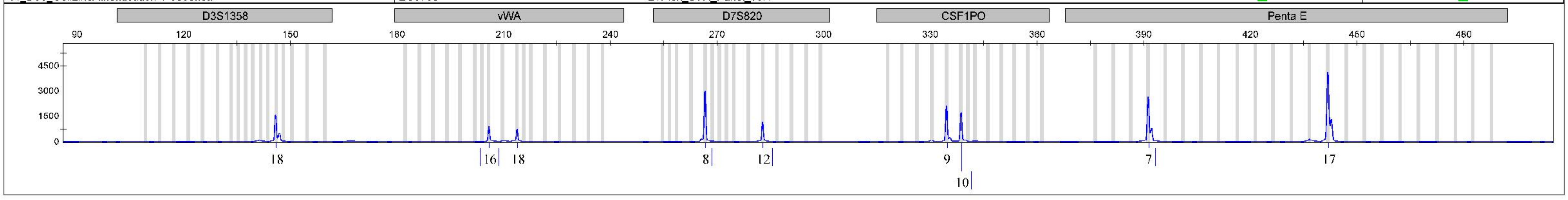

Sample File	Sample Name	Panel
44_D06_CellLineAthentication-1-0530.fsa	EC9706	21Plex_STR_Panel_v0.4

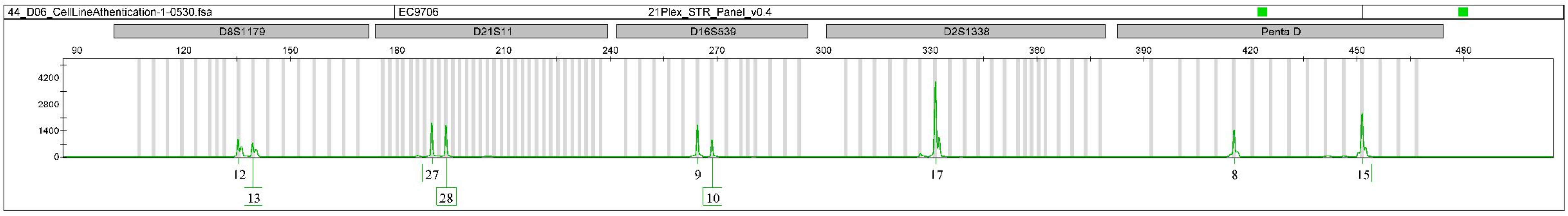

Sample File	Sample Name	Panel
44_D06_CellLineAthentication-1-0530.fsa	EC9706	21Plex_STR_Panel_v0.4

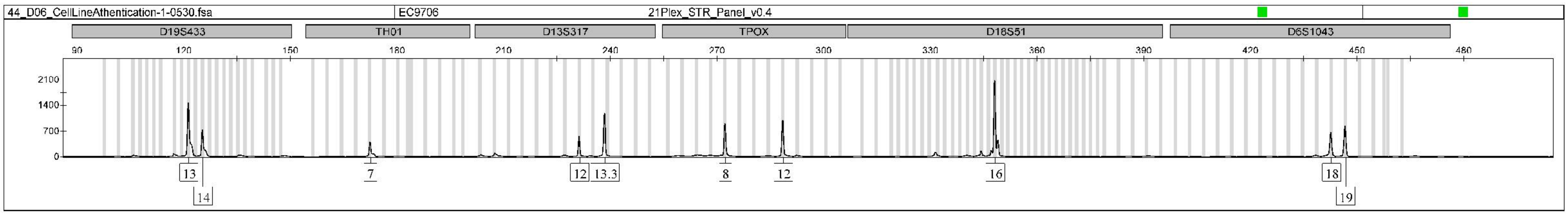

Sample File	Sample Name	Panel
44_D06_CellLineAthentication-1-0530.fsa	EC9706	21Plex_STR_Panel_v0.4

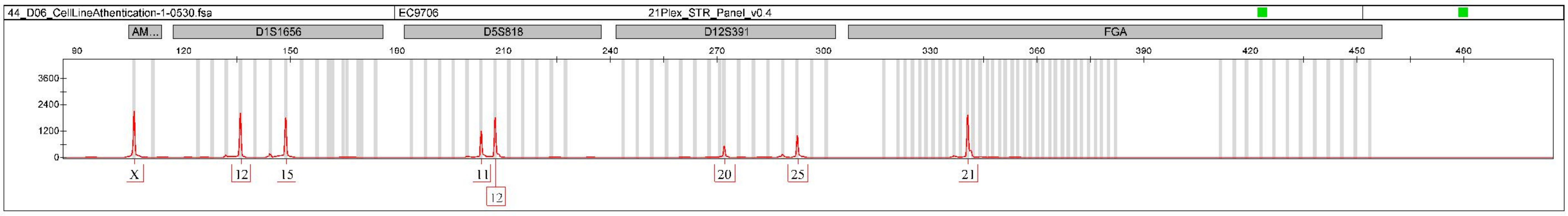

细胞 STR 检测报告

一、检测样品

样品名称：ECA-109

样品编号：BNCC 337687

二、检测项目：STR 基因型检验

三、检测方法：用 Axygen 的基因组抽提试剂盒提取 DNA，采用 21-STR 扩增方案扩增，在 ABI 3730XL 型遗传分析仪上对 STR 位点和性别基因 Amelogenin 进行检测。

四、检验结果：

1，检验基本情况

编号	多等位基因	匹配细胞系	细胞库	EV 值	匹配说明
337687	有		DSMZ		无匹配

多等位基因指三等位及以上基因现象。

本次检测各细胞分型结果良好。

2，样本描述

BNCC 337687：该细胞系未发现交叉污染，为国内建系细胞，因数据库未收录细胞系 ECA-109 相关 STR 数据信息，无匹配结果，若为发表论文用，可提交该 STR 数据。

本次检测在该细胞系中发现多等位基因。

备注：待测细胞系与收录于 ATCC, DSMZ, JCRB 和 RIKEN 等数据库的细胞系 STR 数据进行比对，未收录于以上细胞库的细胞系将无法匹配。

附表：ECA-109 细胞的 STR 位点和 Amelogenin 位点的基因分型结果

ECA-109 细胞			
Marker	Allele 1	Allele 2	Allele 3
D5S818	11	12	
D13S317	10	12	13.3
D7S820	10	12	
D16S539	9	10	
VWA	14	16	17
TH01	6	7	9
AMEL	X	X	
TPOX	8	9	12
CSF1PO	10	11	
D12S391	20	21	
FGA	18	21	
D2S1338	17	23	
D21S11	30	30	
D18S51	13	16	
D8S1179	12	12	
D3S1358	15	18	

其他说明

分型方案及位点分布

	方案 1	方案 2	方案 3	方案 4
1	D3S1358	D8S1179	D19S433	AMEL
2	VWA	D21S11	TH01	D1S1656
3	D7S820	D16S539	D13S317	D5S818
4	CSF1PO	D2S1338	TPOX	D12S391
5	PENTAE	PENTAD	D18S51	FGA
6			D6S1043	

签发日期：2019.6.17

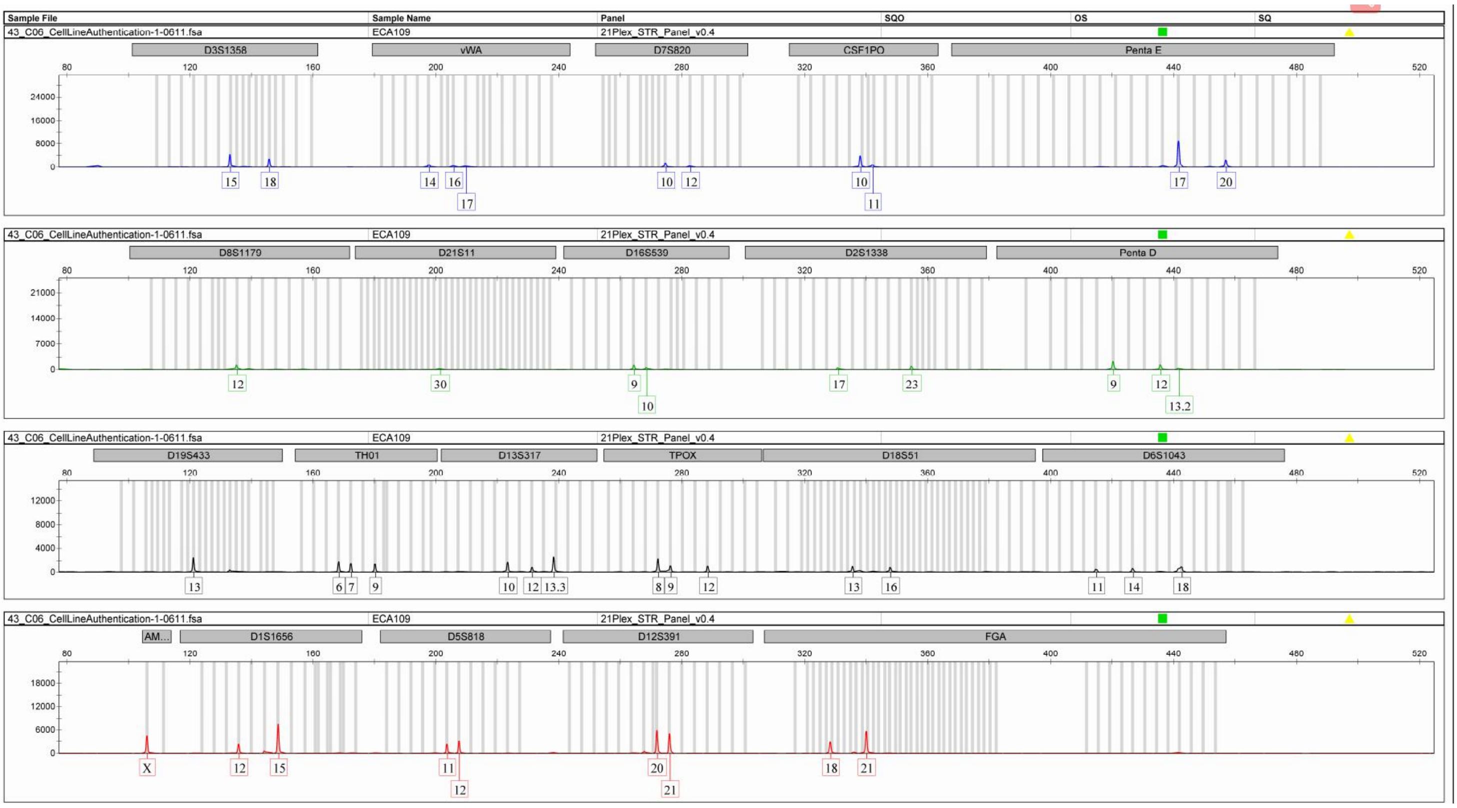
Sample File
Sample Name
Panel
SQO
OS
SQ
43_C06_CellLineAuthentication-1-0611.fsa
ECA109
21Plex_STR_Panel_v0.4
D3S1358
vWA
D7S820
CSF1PO
Penta E
15 18 14 16 17 10 12 10 11 17 20
D8S1179
D21S11
D16S539
D2S1338
Penta D
12 30 9 10 17 23 9 12 13.2
D19S433
TH01
D13S317
TPOX
D18S51
D6S1043
13 6 7 9 10 12 13.3 8 9 12 13 16 11 14 18
AM...
D1S1656
D5S818
D12S391
FGA
X 12 15 11 12 20 21 18 21
