# Supplementary material for: USP36 facilitates esophageal squamous carcinoma progression via stabilizing YAP
Source: Cell Death Dis. 2022 Dec 5;13(12):1021. doi: 10.1038/s41419-022-05474-5 (PMC9722938; doi:10.1038/s41419-022-05474-5)
Supplement: Supplementary file 9 — Language certificate [file 41419_2022_5474_MOESM9_ESM.pdf]

SPRINGER NATURE

Author Services

Editing Certificate

This document certifies that the manuscript

USP36 facilitates Hippo/YAP axis and esophageal squamous carcinoma progression via stabilizing YAP

prepared by the authors

Wenhao Zhang1*, Junwen Luo1, Zhaohua Xiao1, Yifeng Zang2, Xin Li3, Youjia Zhou1, Jie Zhou1, Zhongxian Tian1,5, Jian Zhu2,4# and Xiaogang Zhao1,5#

was edited for proper English language, grammar, punctuation, spelling, and overall style by one or more of the highly qualified native English speaking editors at SNAS.

This certificate was issued on **July 5, 2022** and may be verified on the SNAS website using the verification code **BC38-8044-EFBA-3FFE-9F8P**.

Neither the research content nor the authors' intentions were altered in any way during the editing process. Documents receiving this certification should be English-ready for publication; however, the author has the ability to accept or reject our suggestions and changes. To verify the final SNAS edited version, please visit our verification page at secure.authorservices.springernature.com/certificate/verify.
If you have any questions or concerns about this edited document, please contact SNAS at support@as.springernature.com.

SNAS provides a range of editing, translation, and manuscript services for researchers and publishers around the world.
For more information about our company, services, and partner discounts, please visit authorservices.springernature.com.
