# Supplementary material for: USP36 facilitates esophageal squamous carcinoma progression via stabilizing YAP
Source: Cell Death Dis. 2022 Dec 5;13(12):1021. doi: 10.1038/s41419-022-05474-5 (PMC9722938; doi:10.1038/s41419-022-05474-5)
Supplement: Supplementary file 10 — Ethnic approvement [file 41419_2022_5474_MOESM10_ESM.pdf]

山东大学第二医院科研伦理委员会
批　准　书

批准号：KYLL-2020(LW)-083

论文题目：Deubiquitinase USP36 inhibits HIPPO/YAP signaling and promotes esophageal squamous cell carcinoma progression

第一作者：张文豪　　所在单位：山大二院　　联系电话：15064112032

通讯作者：赵小刚　　所在单位：山大二院　　联系电话：17660080007

拟投稿期刊：Cell death & disease

评审意见：

论文“Deubiquitinase USP36 inhibits HIPPO/YAP signaling and promotes esophageal squamous cell carcinoma progression”

经伦理委员会审查：

研究者的资格、经验符合试验要求；研究方案符合科学性和伦理原则的要求；获得知情同意的方法适当；受试者可能遭受的风险程度与研究预期的受益相比合适。

同意开展该项研究。

山东大学第二医院科研伦理委员会

主任委员：新王印传

山东大学第二医院科研伦理委员会 3701057265328

年　　月　　日

Research ethics committee approval of the Second Hospital of Shandong university

Approval number： KYLL-2020(LW)-083

TITLE：Deubiquitinase USP36 inhibits HIPPO/YAP signaling and promotes esophageal squamous cell carcinoma progression

First author：Wenhao Zhang   **Institute for the study： The Second Hospital, Cheeloo College of Medicine, Shandong University**

Corresponding author： Xiaogang Zhao   **Institute for the study：The Second Hospital, Cheeloo College of Medicine, Shandong University**

Periodicals to be contributed： Cell death & disease

Review comments：

TITLE: Deubiquitinase USP36 inhibits HIPPO/YAP signaling and promotes esophageal squamous cell carcinoma progression

Review by the ethics committee：

The qualification and experience of researcher meet the test requirements; the research project is accordance with the scientific and ethical principles; the method of obtaining informed consent is right; the patients can get more expected benefits compared to the risk degree;.

The study was approved by the Ethics Committee of the second hospital of Shandong university.

Research Ethics committee of the second hospital of shandong university

Chairman of Committee：

Date:

山东大学第二医院科研伦理委员会

批　准　书

批准号：KYLL-2021(LW)093

论文题目：Deubiquitinase USP36 inhibits HIPPO/YAP signaling and promotes esophageal squamous cell carcinoma progression

第一作者：张文豪　　所在单位：山大二院　　联系电话：15064112032

通讯作者：赵小刚　　所在单位：山大二院　　联系电话：17660080007

拟投稿期刊：Cell death & disease

评审意见：

论文“Deubiquitinase USP36 inhibits HIPPO/YAP signaling and promotes esophageal squamous cell carcinoma progression”

经伦理委员会审查：

该项目必须用实验动物进行研究，实验相关人员资格和实验相关单位合适，实验所用动物的品种品系、质量等级、规格基本合适；研究方法符合常规，实验设计基本符合有关动物保护原则，实验动物福利伦理等伦理要求，申请者承诺自觉遵守有关实验动物伦理、随时接受本委员会的监督与检查。

同意开展该项目的研究。

山东大学第二医院科研伦理委员会

主任委员：新王印传

山东大学第二医院科研伦理委员会 3701057265323

年　　月　　日

Research ethics committee approval of the second hospital of Shandong university

Approval number: KYLL-2021(LW)093

TITLE: Deubiquitinase USP36 inhibits HIPPO/YAP signaling and promotes esophageal squamous cell carcinoma progression

First author: Wenhao Zhang Institute for the study: The Second Hospital, Cheeloo College of Medicine, Shandong University

Corresponding author: Xiaogang Zhao Institute for the study: The Second Hospital, Cheeloo College of Medicine, Shandong University

Periodicals to be contributed: Cell death & disease

Review comments:

TITLE: Deubiquitinase USP36 inhibits HIPPO/YAP signaling and promotes esophageal squamous cell carcinoma progression

Review by the ethics committee:

The project must be studied in laboratory animals. Experiment related personnel qualification and experiment related units are appropriate. Varieties, quality grade and specifications of animals used in experiments are appropriate. The experimental design is in accordance with the principles of animal protection, experimental animal welfare ethics and other ethical requirements, Applicants are committed to abide by the relevant experimental animal ethics, and accept the supervision and inspection of the Committee at any time.

We agree to this research of the project.

Research ethics committee of the second hospital of shandong university

Chairman of Committee:

Date:
